# *Nitrogeniibacter aestuarii* sp. nov., a Novel Nitrogen-Fixing Bacterium Affiliated to the Family *Zoogloeaceae* and Phylogeny of the Family *Zoogloeaceae* Revisited

**DOI:** 10.3389/fmicb.2021.755908

**Published:** 2021-10-20

**Authors:** Zhaobin Huang, Renju Liu, Fenghua Chen, Qiliang Lai, Aharon Oren, Zongze Shao

**Affiliations:** ^1^College of Oceanology and Food Science, Quanzhou Normal University, Quanzhou, China; ^2^Key Laboratory of Inshore Resources Biotechnology (Quanzhou Normal University), Fujian Province University, Quanzhou, China; ^3^Key Laboratory of Marine Genetic Resources, Third Institute of Oceanography, Ministry of Natural Resources, Xiamen, China; ^4^The Institute of Life Sciences, The Hebrew University of Jerusalem, Edmond J. Safra Campus, Jerusalem, Israel

**Keywords:** *Nitrogeniibacter*, *Zoogloeaceae*, nitrogen fixation, polyphasic taxonomy, phylogenomic tree

## Abstract

Members of the family *Zoogloeaceae* within the order *Rhodocyclales* are found to play vital roles in terrestrial and aquatic ecosystems by participating in biofloc formation in activated sludge, polycyclic aromatic hydrocarbon degradation, and nitrogen metabolism, such as denitrification and nitrogen fixation. Here, two bacterial strains designated H1-1-2A^T^ and ZN11-R3-1 affiliated to the family *Zoogloeaceae* were isolated from coastal wetland habitats. The 16S rRNA gene sequences of the two strains were 100% identical and had maximum similarity with *Nitrogeniibacter mangrovi* M9-3-2^T^ of 98.4% and ≤94.5% with other species. Phylogenetic analysis suggested that the two strains belonged to a single species and formed a novel monophyletic branch affiliated to the genus *Nitrogeniibacter*. The average nucleotide identity (ANI) value and digital DNA-DNA hybridization (dDDH) estimate between the two strains and *N. mangrovi* M9-3-2^T^ were 78.5–78.7% and 21.4–21.6%, respectively, indicating that the two strains represent a novel species. The genomes of strain H1-1-2A^T^ (complete genome) and ZN11-R3-1 (draft genome) were 4.7Mbp in length encoding ~4,360 functional genes. The DNA G+C content was 62.7%. Nitrogen fixation genes were found in the two strains, which were responsible for the growth on nitrogen-free medium, whereas denitrification genes found in *N. mangrovi* M9-3-2^T^ were absent in the two strains. The respiratory quinone was ubiquinone-8. The major polar lipids consisted of phosphatidylethanolamine, diphosphatidylglycerol, phosphatidylglycerol, and aminophospholipid. The major fatty acids were summed feature 3 (C_16:1_*ω*7*c* and C_16:1_*ω*6*c*), C_16:0_, C_12:0_, and C_10:0_ 3-OH. Based on genomic, phenotypic, and chemotaxonomic characterizations, strains H1-1-2A^T^ and ZN11-R3-1 represent a novel species of the genus *Nitrogeniibacter*, for which the name *Nitrogeniibacter aestuarii* sp. nov. is proposed. The type strain is H1-1-2A^T^ (=MCCC 1K04284^T^=KCTC 82672^T^), and additional strain is ZN11-R3-1 (=MCCC 1A17971=KCTC 82671). Additionally, phylogenomic analysis of the members of the family *Zoogloeaceae* including type strains and uncultivated bacteria was performed, using the Genome Taxonomic Database toolkit (GTDB-Tk). Combined with the 16S rRNA gene phylogeny, four novel genera, *Parazoarcus* gen. nov., *Pseudazoarcus* gen. nov., *Pseudothauera* gen. nov., and *Cognatazoarcus* gen. nov., were proposed. This study provided new insights to the taxonomy of the family *Zoogloeaceae*.

## Introduction

The family *Zoogloeaceae* as a member of the order *Rhodocyclales* was firstly proposed in 2017 (Boden et al., 2017). Thus far, six genera with validly published names were described,^1^ including *Zoogloea* (Shin et al., 1993), *Azoarcus* (Rabus et al., 2019), *Aromatoleum* (Rabus et al., 2019), *Thauera* (Macy et al., 1993), *Uliginosibacterium* (Weon et al., 2008), and *Nitrogeniibacter* (Liao et al., 2021). Members of this family were found to play vital roles in terrestrial and aquatic habitats by participating in biofloc formation in activated sludge (such as *Zoogloea*; Shin et al., 1993), polycyclic aromatic hydrocarbon (PAH) degradation (*Thauera*; Mechichi et al., 2002), and nitrogen metabolism, such as denitrification (*Thauera* and *Nitrogeniibacter*; Liu et al., 2013; Liao et al., 2021) and nitrogen fixation (*Azoarcus*; Lin et al., 2020).

Previously circumscription of the taxonomy of the family *Zoogloeaceae* depended largely on phylogeny of 16S rRNA gene sequences, and a small number of species were included (Boden et al., 2017). The family *Zoogloeaceae* currently includes nearly 50 species with validly published or effectively published names.^2^ With the advance of next-generation sequencing (NGS) and methods of constructing metagenomic-centric genomes and single-cell genomes used for uncultivated bacteria (Rinke et al., 2013; Parks et al., 2018; Lapidus and Korobeynikov, 2021), a large number of genomes affiliated to the family *Zoogloeaceae* and the order *Rhodocyclales* were obtained and released publically in the Genome portal of GenBank. These genomes were obtained from various habitats including wastewater, soil, sediment, and freshwater (Wang et al., 2020). The genomes of uncultivated *Zoogloeaceae* members expanded our knowledge on their ecological niches and phylogenetic diversity. However, the taxonomic position of several members of the family *Zoogloeaceae* is still controversial. For instance, the genus *Niveibacterium* proposed in the family *Rhodocyclaceae* (Chun et al., 2016) is placed as a member within the *Zoogloeaceae* in the EzBioCloud Database (Yoon et al., 2017a); *Thauera hydrothermalis* GD-2^T^ formed a separate branch on the basis of phylogeny of 16S rRNA gene that were distinct from the type species *T. selenatis* ATCC 55363^T^ (Liao et al., 2021). This may be the result of using a small number of species for phylogenetic analysis based on 16S rRNA gene comparison. Thus, the phylogenetic relationship of the *Zoogloeaceae* members needs to be reconsidered, especially on the basis of genome sequences. The Genome Taxonomic Database (GTDB) is considered to be a reliable tool to define the bacterial taxonomic ranks using 120 conserved concatenated proteins (Parks et al., 2018) and is used in accurate assignment for not only the described species but also for genomes of uncultivated organisms. Thus, the phylogeny of the family *Zoogloeaceae* was revisited in this study based on the use of GTDB tools.

*Nitrogeniibacter*, affiliated to the family *Zoogloeaceae*, is a recently proposed genus, with a single species, *N. mangrovi*. The type strain M9-3-2^T^ (=MCCC 1K03313^T^=JCM 32045^T^) was isolated from an enrichment culture of mangrove sediment (Liao et al., 2021). The genus is circumscribed on the basis of 16S rRNA gene phylogeny and concatenated core genes (phylogenomic tree) and physiological and chemical characteristics (Liao et al., 2021). The cells are Gram stain-negative and show anaerobic and aerobic growth, rod-shaped, oxidase-positive, and catalase-positive. Ubiquinone-8 (Q-8) is the major respiratory quinone, diphosphatidylglycerol, phosphatidylethanolamine, phosphatidylglycerol, phospholipids, and aminophospholipids are major polar lipids, and summed feature 3 (C_16:1_*ω*7*c* and C_16:1_*ω*6*c*), C_16:0_, C_10:0_ 3-OH, C_14:0_, and C_10:0_ are major fatty acids. This genus had the ability of denitrification under both aerobic and anaerobic conditions.

In this study, two isolates designated H1-1-2A^T^ and ZN11-R3-1 were obtained from a sediment sample of a *Spartina alterniflora* wetland and from styrofoam plastics collected from a mangrove, respectively. The isolates were found to have identical 16S rRNA gene sequences and likely represented a novel species of the genus *Nitrogeniibacter* within the family *Zoogloeaceae*. This study aimed to determine the taxonomic status of the two isolates using a polyphasic taxonomic approach. Additionally, the phylogeny of the *Zoogloeaceae* members was elucidated based on the available genomes to further advance the taxonomy of the family.

## Materials and Methods

### Bacterial Isolation and Cultivation

Strains H1-1-2A^T^ and ZN11-R3-1 were isolated from a coastal sediment sample and from an enrichment culture inoculated with coastal styrofoam plastics, respectively. The sediment sample was collected from a *Spartina alterniflora* growing area in a wetland (24°86' N, 118°68' E) in Quanzhou Bay, Quanzhou, PR China, on September 05, 2019. A water-extracted medium (WEM) prepared using the nutrients extracted from the sediment with pure water (w/v=1:1) was used to isolate strain H1-1-2A^T^ (Huang et al., 2020b). The 0.2g sediment sample was subjected to 10-fold serial dilutions and spread on the WEM plates and incubated for 2weeks at 28°C. Strain H1-1-2A^T^ was picked and then streaked onto Marine Broth 2216 (MB, BD) agar plates to obtain a pure culture. For the isolation of strain ZN11-R3-1, styrofoam plastic was collected from a mangrove preservation area (24^o^27' N, 117^o^53' E) in Longhai, Zhangzhou, PR China, on November 23, 2019. The plastics were placed into an enrichment medium of 300ml sterile MMC (NaCl 24g/L; MgSO_4_·7H_2_O 7.0g/L; NH_4_NO_3_ 1g/L; KCl 0.7g/L; KH_2_PO_4_ 2.0g/L; and Na_2_HPO_4_·12H_2_O 3.0g/L, pH=7.4) and maintained at 150rpm shaking at 28°C for 2months. An aliquot (2ml) of enriched culture was then transferred to another 100ml fresh MMC medium containing sterile plastics and cultured for another 2months. Then, the enrichment was repeated as above. The biomass in the third enrichment culture was collected using centrifugation at 6,000rpm for 15min and plated on an MB agar plate and maintained at 30°C. Strains H1-1-2A^T^ and ZN11-R3-1 grew well on MB agar plates and MB medium and were stored at −80°C with 20% glycerol (v/v) in the laboratory.

### Phylogeny Analysis Based on 16S rRNA Gene Sequences

The nearly complete 16S rRNA gene sequences of strain H1-1-2A^T^ and strain ZN11-R3-1 were obtained using Sanger sequencing performed as described in a previous study (Huang et al., 2020b). The sequences were also compared with rRNA genes extracted from the genome sequences.

Sequences of the closely related relatives of the two strains were obtained from the EzBioCloud database (Yoon et al., 2017a) and the NCBI nucleotide database.^3^ *Burkholderia cepacia* ATCC 25416^T^ was selected as an outgroup. Then, the 16S rRNA gene sequences were aligned and subjected to phylogenetic analysis using two algorithms, neighbor-joining (NJ) and maximum likelihood (ML) methods with 1,000 bootstraps using MEGA 7.0 (Huang et al., 2019). The best model (T92+G+I) with the lowest Bayesian information criterion (BIC) scores was selected.

### Genome Sequencing and Gene Annotation

The draft genome sequences of strain H1-1-2A^T^ and strain ZN11-R3-1 were determined using the Illumina HiSeq X-Ten platform (Shanghai Majorbio Bio-Pharm Technology Co., Ltd., Shanghai, China). A library of ~400bp fragments was constructed, and paired-end (PE) short reads of ~1 Gb were obtained. The PE reads were firstly trimmed to remove the low base of quality <20 and length <50bp using sickle.^4^ Then, clean reads were assembled into contigs using SPAdes v 3.8.0 with a serial of *k* values of 21, 33, 55, 77, 99, 127 and –*careful* flag (Huang et al., 2019). Then, contigs shorter than 1kb were removed from the assembled contigs. The genome quality was evaluated using QUAST (Gurevich et al., 2013).

The complete genome of type strain H1-1-2A^T^ was obtained using PacBio sequencing with one SMART cell. The 10-kb fragment library was constructed followed the manufacturer’s instructions. The long reads were assembled using the SMRT Link (V6.0.0.47841) of PacBio.

The complete 16S rRNA gene sequence was extracted from the genome sequence using RNAmmer (Lagesen et al., 2007). Genome completeness was evaluated using CheckM v1.0.1 (Parks et al., 2015). Gene annotation was carried out using the RAST server (Aziz et al., 2008) and the KAAS system.^5^ Functional genes with high similarity to close relatives were searched using the blast+ program with e-value cutoff of 1e-5 (Camacho et al., 2009).

The average nucleotide identity (ANI) values were estimated using OrthoANI computation on the EzBioCloud Database (Yoon et al., 2017b). Digital DNA-DNA hybridization (dDDH) estimates were calculated on the GGDC website.^6^ Average amino acids identity among genomes was calculated using CompareM v0.1.2.^7^ The percentage of conserved proteins (POCP), proposed as genus boundary values was also calculated for genomic comparison (Qin et al., 2014).

### Phylogenomic Analysis

The genomes affiliated to the order *Rhodocyclales* were downloaded from the genome portal in NCBI.^8^ A total of 303 genomes were obtained (until Feb.19, 2021), and the genome quality was checked using CheckM v1.0.1 (Parks et al., 2015). Genomes of <50% completeness and >10% contamination were removed from the following study. In addition, 9 genomes, identified using GTDB-tk v. 0.3.2 (Chaumeil et al., 2019), did not belong to the order *Rhodocyclales*, and these were removed from the study. Then, the phylogenomic tree of the genomes was inferred using a concatenated alignment of 120 bacterial single-copy genes with GTDB-tk v. 0.3.2 by using FastTree (Parks et al., 2018). The tree was edited using the Interactive Tree of Life (iTOL) online (Letunic and Bork, 2007). In addition, a phylogenomic tree based on the genomes of type strains belonging to the order *Rhodocyclales* was also constructed using GTDB-Tk.

### Phenotypic Properties

Gram staining was carried out using a Gram staining kit (Hangzhou Tianhe Microorganism Reagent, Co., Ltd.). Colony morphology was recorded on a MB agar plate after incubation at 30°C for 3days. Catalase activity was tested by using 3% H_2_O_2_ solution. Oxidase activity was tested using the oxidase reagent (1% aqueous solution of N,N,N',N'-tetramethyl-p-phenylenediamine dihydrochloride, bioMérieux, France). Motility was observed by puncturing the cells into 0.5% agar. Growth under the anaerobic condition was tested by inoculating the cells into an anaerobic MB medium for 7days. The growth temperature range, NaCl tolerance range, pH range of the strains, and hydrolysis of substrates were determined as described in our previous study (Huang et al., 2020b). Growth on nitrogen-free medium was tested following the method of Huang et al. with 5g/l NaCl and 5g/l glucose (Huang et al., 2014). *N. mangrovi* M9-3-2^T^ (=MCCC 1K03313^T^), obtained from the Marine Culture Collection Center (MCCC), was used as a reference strain.

Physiological and biochemical characterization was carried out using API ZYM, API 20NE, and API 20E kits according to the manufacturer’s instructions (bioMérieux, France). The tested strains and the reference strain were maintained under identical laboratory conditions. Test strips were maintained at 35°C for determining the physiological and biochemical properties.

### Chemotaxonomic Characteristics

For the analysis of fatty acids composition, the strains and reference strain were cultured in MB at 35°C for 3days and cells were collected by centrifugation at 8,000rpm for 10min. The cellular fatty acids were saponified, methylated and extracted, and then identified following the standard MIDI protocol (Sherlock Microbial Identification System, version 6B).

For the polar lipids analysis, strain H1-1-2A^T^ was cultured in MB medium at 35°C for 3days, and cells were harvested by using centrifugation as above. Polar lipids were extracted using a chloroform/methanol system and analyzed using one- and two-dimensional TLC using Merck silica gel 60F254 aluminum-backed thin-layer plates. Lipids were detected and identified by spraying the specific reagents (Huang et al., 2020a).

## Results and Discussion

### Phylogeny of 16S rRNA Gene Sequences

The 16S rRNA gene sequences of strains H1-1-2A^T^ and ZN11-R3-1, obtained by Sanger sequencing or extracted from the genome sequences, had 100% identity, indicating the two strains belonged to same species. The BOX-PCR genotypic fingerprinting profiles of two strains were similar but distinctive (Supplementary Figure 1), which confirmed that they were not clonal. Also, the fingerprinting of the two strains were totally different from *N. mangrovi* M9-3-2^T^, indicating they may belong to a novel species different from *N. mangrovi*.

Sequence similarity search showed that the 16S rRNA gene sequence of strain H1-1-2A^T^ had the maximum similarity (99.6%) with an uncultured bacterium clone IWNB003 (accession number: FR744543), followed by *N. mangrovi* M9-3-2^T^ (98.4%), and had sequence similarities of ≤94.5% with other species affiliated to the family *Zoogloeaceae*. The clone IWNB003 was found in nitrate-amended injection seawater from an oil field (Gittel et al., 2012), and *N. mangrovi* M9-3-2^T^ has the ability of denitrification (Liao et al., 2021), which may indicate that *Nitrogeniibacter* members play valuable roles in nitrogen cycle in the environment.

**Figure 1:**
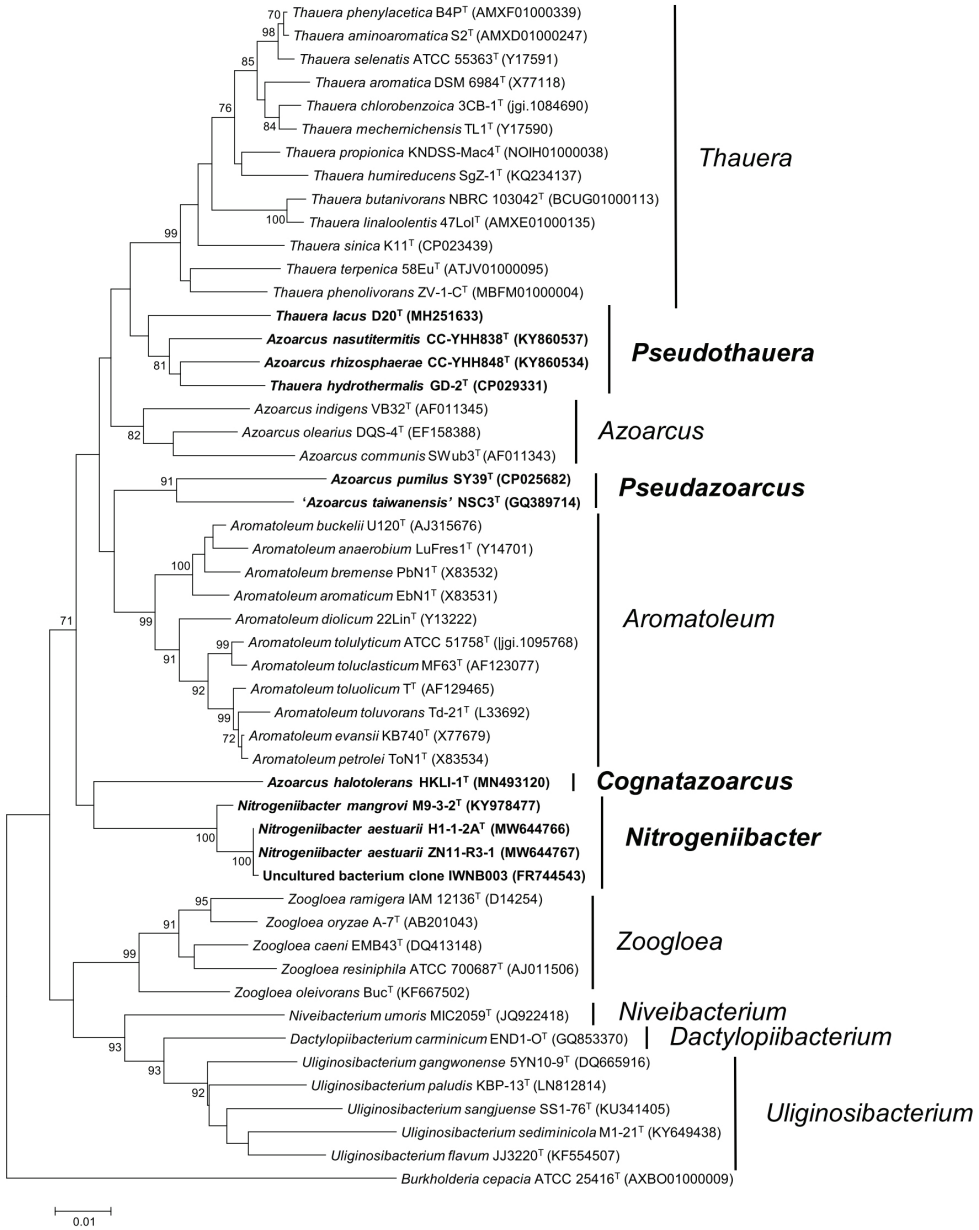
Phylogeny of 16S rRNA gene sequences. The tree was constructed using the neighbor-joining method. Bootstrapping was carried out with 1,000 replicates. Branch node values below 70% are not shown. *Burkholderia cepacia* ATCC 25416^T^ (AXBO01000009) was selected as the outgroup. Bar, 0.01 represented the nucleotide substitution per position. Members of *Nitrogeniibacter*, *Pseudazoarcus*, and *Pseudothauera* are marked bold.

Phylogeny of 16S rRNA gene sequence inferred from the ML and NJ methods placed strains H1-1-2A^T^ and ZN11-R3-1 within the genus *Nitrogeniibacter* as a novel monophyletic line, distinct from *N. mangrovi* M9-3-2^T^. This indicated that the two strains could be considered as a novel species of the genus *Nitrogeniibacter* (Figure 1; Supplementary Figure 2).

Phylogeny of 16S rRNA gene sequences indicated that the members of *Azoarcus* and the members of *Thauera* were separated into different clades, which were clearly separated from the type species, *A. indigens* and *T. selenatis*. Firstly, *A. pumilus* SY39^T^ and “*A*. *taiwanensis*” NSC3^T^ formed a separate cluster, which did not cluster with the type species *A. indigens*. Here, we named this cluster as a novel genus *Pseudazoarcus*, which was equal to the group name “Azoarcus_D” of the Genome Taxonomy Database (GTDB; Chaumeil et al., 2019). Thus, *A. pumilus* should be transferred into the genus *Pseudazoarcus*. *Azoarcus pumilus* was designated the type species of this genus and was renamed as *Pseudazoarcus pumilus* comb. nov. “*A*. *taiwanensis*” (a name effectively but not validly published; Lee et al., 2014) was also affiliated to this genus. Secondly, in the phylogenetic clade of the genus *Thauera*, there were four species, including *T. lacus*, *T. hydrothermalis*, *A. nasutitermitis,* and *A. rhizosphaerae* that formed a monophyletic cluster. Though this cluster formed a node with other *Thauera* members, bootstrap support was low (<70% of both ML and NJ; Figure 1; Supplementary Figure 2). The four species may be assigned to a new genus named *Pseudothauera*, which is equivalent to “Thauera_A” in the GTDB taxonomy. Thus, the four species, *T. lacus*, *T. hydrothermalis*, *A. nasutitermitis,* and *A. rhizosphaerae,* should be transferred to a novel genus *Pseudothauera* and renamed as *Pseudothauera lacus* comb. nov., *Pseudothauera hydrothermalis* comb. nov., *Pseudothauera nasutitermitis* comb. nov., and *Pseudothauera rhizosphaerae* comb. nov., respectively. Thirdly, *A. halotolerans* HKLI-1^T^ formed an independent line on the phylogenomic tree, which clearly branched with *Azoarcus*. This species should be reclassified into a novel genus; *Cognatazoarcus halotolerans* gen. nov., comb. nov. was therefore proposed. Fourthly, *A. communis* SWub3^T^ did not cluster together with the type species *A. indigens* and should be transferred into a novel genus. Here, we named this cluster as a novel genus *Parazoarcus*, which was equal to the genus name “Azoarcus_C” of the Genome Taxonomy Database (GTDB).

### Genomic Characteristics

The complete genome of strain H1-1-2A^T^ included one chromosome (4,678,511bp) and one plasmid (66,515bp). The draft genome size of strain ZN11-R3-2 was 4,656,485bp on 42 contigs (>1kb) with N50 value of 343,918bp (Table 1). Three copies of *rrn* operon (16S-

**Table 1:** Differential characteristics of strain H1-1-2A^T^ and strain ZN11-R3-1 compared to the close relative *N. mangrovi* M9-3-2^T^.

Characteristics	H1-1-2A^T^	ZN11-R3-1	M9-3-2^T^
Temperature (optimum, ^o^C)	15–40 (35)	15–40 (35)	25–40 (35)
pH	7–8	7–8	7
Alkaline phosphatase	+	+	w
Lipase (C14)	−	−	w
Reduction of nitrate to nitrite	−	−	+
Trisodium citrate as sole carbon source for growth	+	+	−
Voges-Proskauer reaction	−	−	w
Genome size (bp, >1kb)	4,745,026	4,656,485	4,236,644
Functional genes	4,364	4,317	3,884
DNA G+C content (%)	62.67	62.71	67.13
Isolation source	*Spartina alterniflora* sediment	Plastics in mangrove sediment	Mangrove sediment

All strains were positive for leucine arylamidase, weak positive for esterase (C4), valine arylamidase, acid phosphatase, naphthol-AS-BI-phosphohydrolase. Hydrolysis of aesculin were weak positive. +, positive; w, weak positive; −, negative.

23S-5S rRNA genes) were found in the complete genome, and 16S rRNA gene copies were 1,528bp in length and had 100% identity. The genome size of the strains was a little larger than *N. mangrovi* M9-3-2^T^ (a chromosome of 4,236,644bp; accession number: CP048836). The DNA G+C content of strains H1-1-2A^T^ and ZN11-R3-1 were 62.67 and 62.71%, respectively, which were a little lower than *N. mangrovi* M9-3-2^T^ (67.13%). Gene prediction showed that there were 4,364 and 3,884 predicted genes in strains H1-1-2A^T^ and M9-3-2^T^, respectively. dDDH and ANI values between strain H1-1-2A^T^ and ZN11-R3-1 were estimated to be 85.6 and 98.4%, respectively. These values exceeded the threshold of species delineation, which strongly supported that the two strains belonged to the same species (Kim et al., 2014). dDDH and ANI values between strains H1-1-2A^T^ and ZN11-R3-1 and *N. mangrovi* M9-3-2^T^ were 21.4–21.6% and 78.50–78.72%, indicating that strains H1-1-2A^T^ and ZN11-R3-1 represented a novel species.

Strains H1-1-2A^T^ and ZN11-R3-1 contained nitrogen fixation gene clusters encoding nitrogenase reductase (*nifH*, KO list: K02588), nitrogenase molybdenum-iron protein (*nifD*, K02586; *nifK*, K02591), and related proteins (*modABCD*; Supplementary Table 1), which were also found in *N. mangrovi* M9-3-2^T^ (Liao et al., 2021). The nitrogen fixation genes were assumed to enable their growth on nitrogen-free medium. Interestingly, denitrification genes, *nirBD* (K000362 and K000363), *norBC* (K004561 and K002305), and *narGHIJ* (K000370, K000371, K000373, and K000374), were not found in strains H1-1-2A^T^ and ZN11-R3-1, but they were present in *N. mangrovi* M9-3-2^T^ (Supplementary Table 2), in which denitrification was confirmed in a laboratory experiment (Liao et al., 2021). In addition, the *sox* system (*soxABCDXYZ* gene cluster) was present in strain H1-1-2A^T^ (Supplementary Table 3) and in *N. mangrovi* M9-3-2^T^ (Liao et al., 2021), suggesting that *Nitrogeniibacter* members may have the ability of sulfur oxidation.

### Phylogenomics of the Family *Zoogloeaceae*

**Figure 2:**
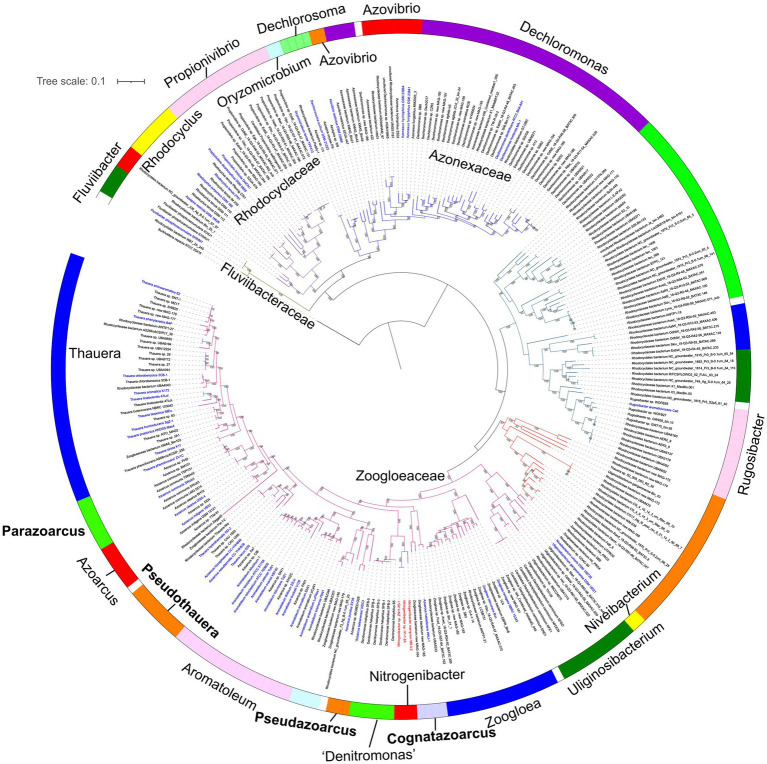
Phylogenomic analysis based on 120 bacterial covered single-copied gene sets of the members affiliated to the order *Rhodocyclales* using FastTree. The bootstrap values on the node are displayed by >70. Bar, 0.1 represents the nucleotide substitutions per position. The blue names represent validly published species. Red names showed *Nitrogeniibacter* members. The genus names were shown around the color circle. Four genera proposed in this study are marked bold.

The development of MAG binning and single-cell genomes contributed large numbers of genome sequences of uncultivated bacteria, including members of family *Zoogloeaceae* and the order *Rhodocyclales*, which could expand knowledge on the phylogenetic diversity based on core genome analysis. Here, the genomes of the order *Rhodocyclales* with ≥50% completeness and≤10% contamination were used, of which the genome quality was verified to perform accurate phylogenetic analysis by GTDB-Tk (Bowers et al., 2017). A total of 277 genomes affiliated to the order *Rhodocyclales* that meet the above standards were used in the phylogenomic analysis. Compared to the 78 and 92 genomes analyzed in phylogenomic studies of the order *Rhodocyclales* by Wang et al. (2020) and Liao et al. (2021), respectively, our study further expanded the known phylogenetic groups within the order *Rhodocyclales*. The described species account for a minor part of the phylogenomic tree, indicating that majority of the members of *Rhodocyclales* are still waiting to be cultivated (Figure 2).

Phylogenomic analysis based on 120 bacterial conserved single-copy genes strongly placed strains H1-1-2A^T^ and ZN11-R3-1 in a sister group of the genus *Nitrogeniibacter*, which was neighbored by “*Denitromonas*.” This agreed with the phylogeny based on concatenated core genome sequences (Liao et al., 2021). “*Denitromonas*” should be transferred into the family *Zoogloeaceae* and did not belong to the family *Rhodocyclaceae*.^9^ In the lineages of the family *Zoogloeaceae*, the relationship between *A. pumilus* SY39^T^ and “*A. taiwanensis*” NSC3^T^ showed congruent topology with 16S rRNA gene phylogeny (Figure 1), which strongly supported the two species should be reclassified into a novel genus, for which we propose the name *Pseudazoarcus*. Also, in the phylogenomic tree, *T. lacus* D20^T^, *T. hydrothermalis* GD-2^T^, *A. nasutitermitis* CC-YHH838^T^, and *A. rhizosphaerae* CC-YHH848^T^ formed a monophyletic cluster, which also supported the phylogeny of the 16S rRNA gene. The four species should be assigned to a new genus, for which we propose the name *Pseudothauera*. In addition, phylogenomic analysis of *A. olearius* DQS-4^T^, *A. indigens* VB32^T^, and *A. communis* SWub3^T^ showed topology incongruent with the 16S rRNA gene, possibly due to the small number of sequences used. Thus, it is proposed that *A. communis* SWub3^T^ be reclassified into a novel genus named *Parazoarcus* gen. nov. *Azoarcus halotolerans* HKLI-1^T^, which is only distantly related to the type species *A. indigens*, should also be reclassified into a novel genus. Thus, *Cognatazoarcus* gen. nov. was proposed. *Niveibacterium* firstly proposed in the family *Rhodocyclaceae* (Chun et al., 2016) should be transferred to the family *Zoogloeaceae* based on the phylogenetic analysis. Finally, a family-level lineage including the genus *Rugosibacter* was clearly separated from the family *Zoogloeaceae*, indicating that *Rugosibacter* may represent a novel family.

**Figure 3:**
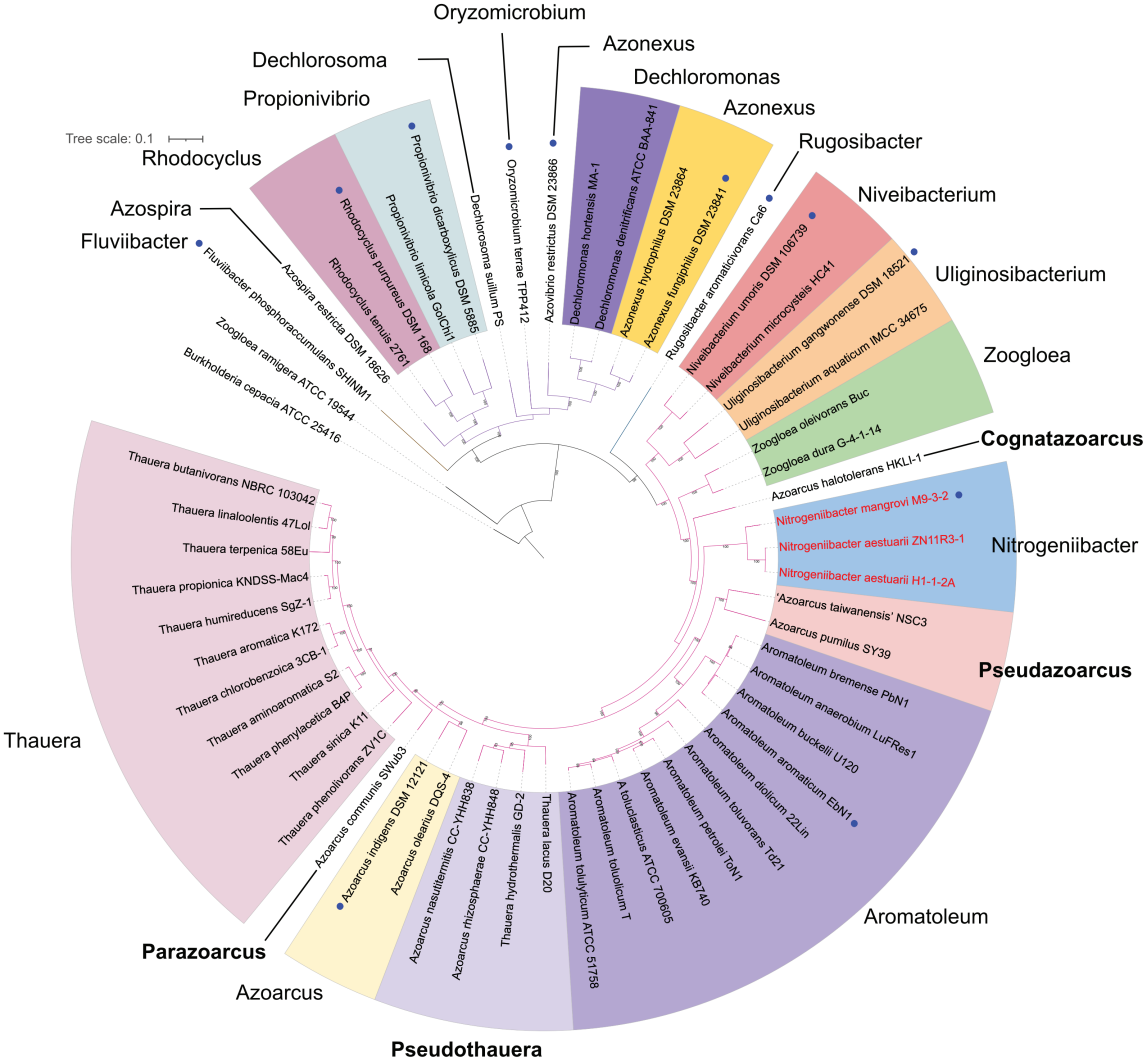
Phylogenomic analysis of the *Nitrogeniibacter* members and type strains affiliated to the order *Rhodocyclales* based on 120 bacterial conserved single-copied gene sets of the members. The bootstrap values on the nodes are displayed by >70. Bar, 0.1 represents the nucleotide substitution per position. Blue circles represent type species. Red names show *Nitrogeniibacter* members. The branch color represents the families, *Zoogloeaceae*, *Azonexaceae*, and *Rhodocyclaceae* of the order *Rhodocyclales*. Four genera proposed in this study are marked bold.

**Figure 4:**
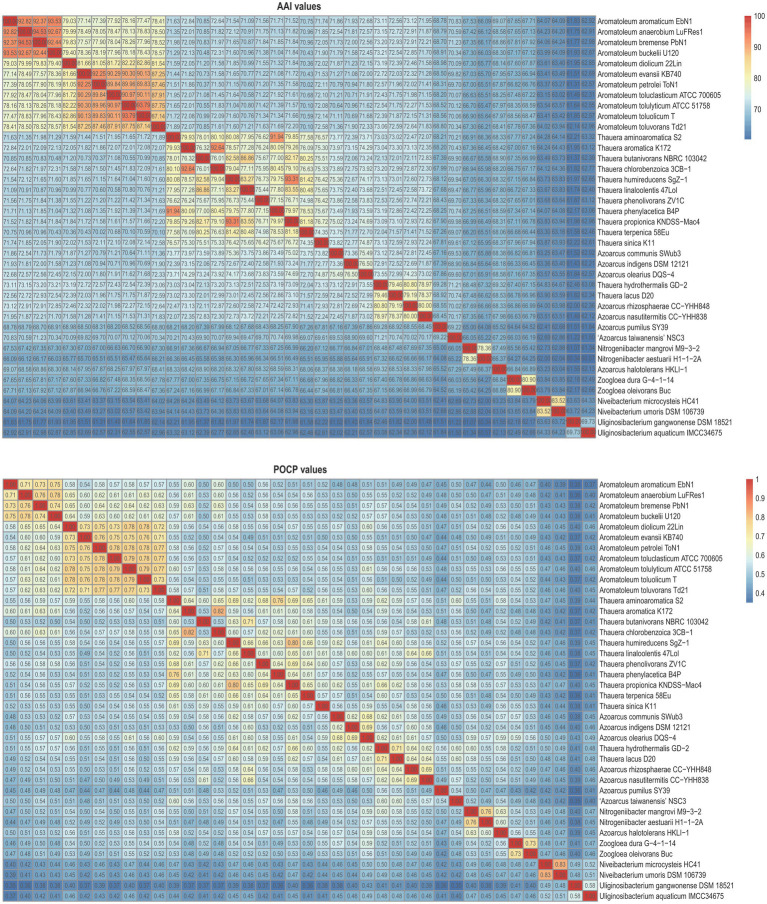
Heatmap showing the AAI values **(upper panel)** and POCP values **(lower panel)** among the members of the family *Zoogloeaceae*.

Figure 3 presents a small phylogenomic tree reconstructed using GTDB-tk, only including the type strains. Two genomes, *Thauera selenatis* AX^T^ (type species) with high genome contamination and “*Zoogloea ramigera*” ATCC 19544, possibly incorrectly named, were excluded (Supplementary Table 4). The topology of the small tree was congruent with that of the large tree, which supported the above analysis. AAI values calculated among the 40 members of the family *Zoogloeaceae* ranged from 60.34 to 94.53% (Figure 4), which exceeded the family boundary of >45% (Konstantinidis et al., 2017). Thus, the members should be considered to belong to the family *Zoogloeaceae*. Our analysis did not support the proposal of *Uliginosibacterium* as an independent family (Wang et al., 2020). Compared to POCP values, AAI values demonstrated certain advantages to delineate the genus boundary of the members of the *Zoogloeaceae* (Figure 4). The calculation of POCP values depends on the similarity of the protein contents of genomes, which had similar genome size (Qin et al., 2014). It is reported that POCP values are also not effective and appropriate for delineating the genera of the families *Acetobacteraceae* (Rai et al., 2021), *Rhodobacteraceae* (Suresh et al., 2019), and *Methylococcaceae* (Orata et al., 2018). For instance, the four species, *T. lacus*, *T. hydrothermalis*, *A. nasutitermitis*, and *A. rhizosphaerae,* clearly grouped together, ranging from 78.37 to 80.80% of the AAI values for the type strains, which were below the recommended genus cutoff of <80% (Luo et al., 2014). The four species were distinctly separated from *Thauera* members and other genera (Figure 4). The AAI values of *Nitrogeniibacter* compared to the genera *Thauera*, *Parazoarcus*, *Azoarcus*, *Pseudothauera*, *Pseudazoarcus*, and *Cognatazoarcus* were 65.5–67.4%, 65.9–66.8%, 65.6–67.0%, 66.2–68.5%, 64.1–66.1%, and 66.4–67.5%, respectively, which were below the genus cutoff of <80% (Luo et al., 2014). Thus, our study expanded the family *Zoogloeaceae* into 11 genera, including *Zoogloea*, *Azoarcus*, *Aromatoleum*, *Thauera*, *Niveibacterium*, *Uliginosibacterium*, *Nitrogeniibacter*, *Parazoarcus*, *Cognatazoarcus*, *Pseudazoarcus*, and *Pseudothauera*.

### Phenotypic Properties

Colonies of strains H1-1-2A^T^ and ZN11-R3-1 on MB agar plates at 30°C were round, transparent, convex, and~1mm in diameter. The cells were rod-shaped, motile, and stained Gram-negative. Catalase activity and oxidase activity were found to be positive, similar to *N. mangrovi* M9-3-2^T^. The tested strains did not degrade soluble starch, skim milk, carboxymethyl cellulose, and Tweens 40, 60, and 80. Anaerobic growth was not observed for strains H1-1-2A^T^ and ZN11-R3-1. The two strains can grow at 15–40°C with the optimum at 35°C and a pH range of 7.0–8.0 (Table 1). NaCl tolerance was observed at 0–4% (w/v) with the optimum of 0.5%, similar to *N. mangrovi* M9-3-2^T^ (Table 1). Strains H1-1-2A^T^ and ZN11-R3-1 can grow on nitrogen-free medium, similar to *N. mangrovi* M9-3-2^T^. Nitrate cannot be reduced by strains H1-1-2A^T^ and ZN11-R3-1, in contrast to *N. mangrovi* M9-3-2^T^ which has denitrification ability. Additional biochemical and physiological properties of strains H1-1-2A^T^ and ZN11-R3-1 are listed in the species description.

### Chemotaxonomic Properties

The respiratory quinone of strain H1-1-2A^T^ was ubiquinone-8 (Q-8), as in the related *N. mangrovi* M9-3-2^T^ and other members of family *Zoogloeaceae* (Liao et al., 2021). The polar lipids consisted of phosphatidylethanolamine (PE), diphosphatidylglycerol (DPG), and phosphatidylglycerol (PG), two unidentified aminophospholipid (APL), one other phospholipid (PL), and one unidentified lipid (L; Supplementary Figure 3). The predominant fatty acids (>5%) of strain H1-1-2A^T^ consisted of summed feature 3 (43.2%), C_16:0_ (23.0%), C_12:0_ (9.5%), and C_10:0_ 3-OH (7.6%), similar to strain ZN11-R3-1 (37.9, 26.1, 6.3, and 5.1%, respectively; Table 2). Although the major fatty acids of strain H1-1-2A^T^ and strain ZN11-R3-1 were similar to *N. mangrovi* M9-3-2^T^, the presence of minor fatty acids such as C_18:0_ showed characteristic differences. The major isoprenoid quinone and major fatty acids of *Nitrogeniibacter* were similar to the closely related genera, *Cognatazoarcus*, *Pseudazoarcus*, *Pseudothauera*, and *Parazoarcus*, and their polar lipids composition showed somewhat different profiles (Supplementary Table 5).

**Table 2:** Fatty acid profile of strain H1-1-2A^T^ and strain ZN11-R3-1 compared to close relative *N. mangrovi* M9-3-2^T^.

Fatty acids	H1-1-2A	ZN11-R3-1	M9-3-2
Saturated			
C_9:0_	2.0	tr	1.1
C_10:0_	2.7	1.0	2.3
C_12:0_	9.5	6.3	2.6
C_14:0_	-	tr	5.1
C_16:0_	23.0	26.1	34.0
C_17:0_	1.5	1.2	1.8
C_18:0_	tr	2.0	6.4
Unsaturated			
iso-C_17:1_ *ω*5*c*	1.0	tr	1.1
C_17:1_ *ω*6*c*	1.1	tr	-
anteiso-C_17:1_ A	-	1.0	tr
iso-C_18:1_ H	1.2	tr	1.1
C_18:1_ *ω*9*c*	-	tr	1.6
Branched			
iso-C_12:0_	-	2.1	tr
Hydroxyl			
C_10:0_ 3-OH	7.6	5.1	5.0
Summed feature 2^†^	-	tr	1.7
Summed feature 3^†^	43.2	37.9	28.7
Summed feature 8^†^	4.7	8.7	4.7

-, not detected; tr, trace (<1%).

† Summed features are groups of two or three fatty acids that cannot be separated by GLC using the MIDI system. Summed feature 2 comprised C_12:0_ aldehyde and unknown 10.9283, summed feature 3 comprised C_16:1_*ω*7*c* and C_16:1_*ω*6*c*, and summed feature 8 comprised C_18:1_ *ω*7*c* and/or C_18:1_ *ω*6*c*.

## Conclusion

Based on the genomic, phylogenetic, phenotypic, and chemotaxonomic characteristics, strains H1-1-2A^T^ and ZN11-R3-1 represent a novel species of the genus *Nitrogeniibacter*, for which the name *Nitrogeniibacter aestuarii* sp. nov. is proposed. The type strain is H1-1-2A^T^ (=MCCC 1K04284^T^=KCTC 82672^T^); ZN11-R3-1 (=MCCC 1A17971=KCTC 82671) is the second strain of the species. Based on the phylogenetic analysis, four novel genera within the family *Zoogloeaceae*, *Parazoarcus* gen. nov., *Pseudothauera* gen. nov., *Pseudazoarcus* gen. nov., and *Cognatazoarcus* gen. nov. were proposed.

### Description of *Nitrogeniibacter aestuarii* sp. nov.

*Nitrogeniibacter aestuarii* (aes.tu.a’ri.i. L. gen. n. *aestuarii*, of a coastal wetland, the source of the type strain isolated from wetland cordgrass and mangrove in estuary).

Colonies on MB agar plates cultured for 3days at 30°C are ~1mm, round, transparent, and convex. Cells are Gram stain-negative and rod-shaped. Growth occurs between 15 and 40°C with an optimum at 35°C, at 0–4% NaCl (w/v) with an optimum of 0.5% and a pH range of 7.0–8.0. Catalase-positive and oxidase-positive. Strains can grow on nitrogen-free medium. Nitrate cannot be reduced to nitrite. Positive for alkaline phosphatase, leucine arylamidase; weakly positive for esterase (C4), valine arylamidase, acid phosphatase, and naphtholAS-BI-phosphohydrolase. Hydrolysis of aesculin is weak positive. Malic acid and trisodium citrate can be used as sole carbon sources.

The quinone system is ubiquinone-8. The major fatty acids are summed feature 3 (C_16:1_*ω*7*c* and C_16:1_*ω*6*c*), C_16:0_, C_12:0_, and C_10:0_ 3-OH. The major polar lipids include phosphatidylethanolamine (PE), diphosphatidylglycerol (DPG), and phosphatidylglycerol (PG). The genome size is 4.7Mbp with DNA G+C content of 62.7%.

The type strain is H1-1-2A^T^ (=MCCC 1K04284^T^=KCTC 82672^T^), isolated from *Spartina alterniflora* wetland sediment. Another strain is ZN11-R3-1 (=MCCC 1A17971=KCTC 82671), isolated from the enrichment culture inoculated with plastics collected from a wetland mangrove.

The GenBank/EMBL/DDBJ accession numbers of 16S rRNA gene sequence of strains H1-1-2A^T^ and ZN11-R3-1 are MW644766 and MW644767, respectively. The whole-genome sequences of strains H1-1-2A^T^ and ZN11-R3-1 have been deposited at GenBank under the accession numbers CP071321-CP071322 and JAFKAB000000000, respectively.

### Emended Description of the Family *Zoogloeaceae*

In addition to the properties listed in the original description (Boden et al., 2017), the family *Zoogloeaceae* includes the genera *Niveibacterium*, *Parazoarcus*, *Pseudothauera*, *Pseudazoarcus*, and *Cognatazoarcus*. The AAI values among the members range from 60.34 to 94.53%. DNA G+C content is 56.6–68.7%.

### Taxonomic Consequences: New Genera

#### Description of *Pseudazoarcus* gen. nov.

*Pseudazoarcus* (Pseud.a.zo.ar΄cus. Gr. masc. adj. *pseudes*, false; N.L. masc. n. *Azoarcus*, a bacterial genus name; N.L. masc. n. *Pseudazoarcus*, false *Azoarcus*).

The description is as that for *Pseudazoarcus pumilus* comb. nov., which is the type species. The genus has been separated from *Azoarcus* based on phylogenetic analyses of 16S rRNA gene and genome sequences. The genomic size is 3.2–4.2Mb. DNA G+C content is 62.8–66.5%.

#### Description of *Pseudothauera* gen. nov.

*Pseudothauera* (Pseu.do.thau΄e.ra. Gr. masc. adj. *pseudes*, false; N.L. fem. n. *Thauera*, a bacterial genus name; N.L. fem. n. *Pseudothauera*, false *Thauera*).

The description is as that for *Pseudothauera hydrothermalis* comb. nov., which is the type species. The genus has been separated from *Thauera* based on phylogenetic analysis of 16S rRNA gene sequence and genome sequences. The genomic size is 3.1Mb-4.7Mb. DNA G+C content is 63.4–68.3%.

#### Description of *Cognatazoarcus* gen. nov.

*Cognatazoarcus* (Cog.nat.a.zo.ar΄cus. L. masc. adj. *cognatus*, relative, related, kindred; N.L. masc. n. *Azoarcus*, a bacterial generic name; N.L. masc. n. *Cognatazoarcus*, related to *Azoarcus*).

The description is as that for *Cognatazoarcus halotolerans* comb. nov., which is the type species. The genus has been separated from *Azoarcus* based on phylogenetic analysis of genome sequences.

#### Description of *Parazoarcus* gen. nov.

*Parazoarcus* (Par.a.zo.ar΄cus. Gr. prep. *para* beside; N.L. masc. n. *Azoarcus*, a bacterial genus name; N.L. masc. n. *Parazoarcus*, beside *Azoarcus*).

The description is as that for *Parazoarcus communis* comb. nov., which is the type species. The genus has been separated from *Azoarcus* based on phylogenetic analysis of genome sequences.

### Taxonomic Consequences: New Combinations for Species

#### Description of *Pseudazoarcus pumilus* comb. nov.

*Pseudazoarcus pumilus* (pu΄mi.lus. L. masc. adj. *pumilus*, small, tiny).

Basonym: *Azoarcus pumilus* Fu et al. 2019.

The description is as for *Azoarcus pumilus* (Fu et al., 2019). The type strain is SY39^T^ (=KCTC 62157^T^=MCCC 1K03430^T^).

#### Description of *Pseudothauera hydrothermalis* comb. nov.

*Pseudothauera hydrothermalis* (hy.dro.ther.ma΄lis. Gr. neut. n. *hydor*, water; Gr. masc. adj. *thermos*, hot; N.L. fem. adj. *hydrothermalis*, hydrothermal).

Basonym: *Thauera hydrothermalis* Yang et al. 2018.

The description is as for *Thauera hydrothermalis* (Yang et al., 2018). The type strain is GD-2^T^ (=NBRC 112472^T^=CGMCC 1.15527^T^).

#### Description of *Pseudothauera lacus* comb. nov.

*Pseudothauera lacus* (la’cus. L. gen. n. *lacus*, of a lake).

Basonym: *Thauera lacus* Zheng et al. 2019.

The description is as for *Thauera lacus* (Zheng et al. 2019). The type strain is D20^T^ (=MCCC 1H00305^T^=KCTC 62586^T^).

#### Description of *Pseudothauera rhizosphaerae* comb. nov.

*Pseudothauera rhizosphaerae* (rhi.zo.sphae’rae. Gr. fem. n. *rhiza*, root; Gr. fem. n. *sphaira*, ball, sphere; N.L. gen. n. *rhizosphaerae*, from the rhizosphere).

Basonym: *Azoarcus rhizosphaerae* Lin et al. 2020.

The description is as for *Azoarcus rhizosphaerae* (Lin et al., 2020). The type strain is CC-YHH848^T^=BCRC 81060^T^=JCM 32002^T^).

#### Description of *Pseudothauera nasutitermitis* comb. nov.

*Pseudothauera nasutitermitis* (na.su.ti.ter’mi.tis. N.L. gen. n. *nasutitermitis*, of a termite of the genus *Nasutitermes*).

Basonym: *Azoarcus nasutitermitis* Lin et al. 2020.

The description is as for *Azoarcus nasutitermitis* (Lin et al., 2020). The type strain is CC-YHH838^T^ (= BCRC 81059^T^=JCM 32001^T^).

#### Description of *Cognatazoarcus halotolerans* comb. nov.

*Cognatazoarcus halotolerans* (ha.lo.to’le.rans. Gr. masc. n. *hals*, *halos*, salt; L. pres. part. *tolerans*, tolerating; N.L. part. adj. *halotolerans*, salt-tolerating).

Basonym: *Azoarcus halotolerans* Lin et al. 2020.

The description is as for *Azoarcus halotolerans* (Li et al., 2020). The type strain is HKLI-1^T^ (= KCTC 72659^T^=CCTCC AB 2019312^T^).

#### Description of *Parazoarcus communis* comb. nov.

*Parazoarcus communis* (com.mu’nis. L. masc. adj. *communis*, usual, common, referring to diverse habitats).

Basonym: *Azoarcus communis* Reinhold-Hurek et al. 1993.

The description is as for *Azoarcus communis* (Reinhold-Hurek et al., 1993). The type strain is SWub3^T^ (= ATCC 51397^T^=DSM 12120^T^=LMG 9095^T^).

## Significance

A novel species named *Nitrogeniibacter aestuarii* with two strains affiliated to the family *Zoogloeaceae* was proposed by using a polyphasic taxonomic approach. The species had the ability of nitrogen fixation, which was assumed to play important roles in the nitrogen cycle of coastal wetlands. Additionally, phylogenetic analysis of the family *Zoogloeaceae* based on genome sequences of type strains and uncultivated bacteria was performed and four novel genera, *Parazoarcus* gen. nov., *Pseudothauera* gen. nov., *Pseudazoarcus* gen. nov., and *Cognatazoarcus* gen. nov., were proposed. This study provided new insights into the taxonomy of the family *Zoogloeaceae*.

## Data Availability Statement

The datasets presented in this study can be found in online repositories. The names of the repository/repositories and accession number(s) can be found in the article/Supplementary Material.

## Author Contributions

ZH and ZS conceived the study and wrote the manuscript. ZH, RL, FC, and QL conducted the experiments. AO proposed names, wrote and checked etymologies, and edited and corrected the manuscript. All authors contributed to the article and approved the submitted version.

## Funding

This work was supported by Marine Microbial Collection Program (2019KJ25) as part of the National Infrastructure of Microbial Resources of China (NIMR 2021-9) and Scientific Research Foundation of Third Institute of Oceanography, Ministry of Natural Resources (2019021).

## Conflict of Interest

The authors declare that the research was conducted in the absence of any commercial or financial relationships that could be construed as a potential conflict of interest.

## Publisher’s Note

All claims expressed in this article are solely those of the authors and do not necessarily represent those of their affiliated organizations, or those of the publisher, the editors and the reviewers. Any product that may be evaluated in this article, or claim that may be made by its manufacturer, is not guaranteed or endorsed by the publisher.

## Footnotes

^1^ https://lpsn.dsmz.de/family/zoogloeaceae

^2^ https://www.ezbiocloud.net/taxonomy?tn=Zoogloeaceae

^3^ https://blast.ncbi.nlm.nih.gov/Blast.cgi

^4^ https://github.com/najoshi/sickle

^5^ https://www.genome.jp/tools/kaas/

^6^ https://ggdc.dsmz.de/

^7^ https://github.com/dparks1134/CompareM

^8^ https://www.ncbi.nlm.nih.gov/genome/

^9^ https://lpsn.dsmz.de/family/rhodocyclaceae

## Supplementary Material

Supplementary Figure 1 Electrophoresis pattern of BOX-PCR fingerprinting of the strains. M. DL 2000 DNA marker; 1, strain M9-3-2^T^; 2, strain H1-1-2A^T^; 3, strain ZN11-R3-1.

Supplementary Figure 2 Phylogeny of 16S rRNA gene sequences. The tree was constructed using the maximum likelihood method. Bootstrapping was carried out with 1,000 replicates. Branch node values below 70% are not shown. *Burkholderia cepacia* ATCC 25416^T^ (AXBO01000009) was selected as the outgroup. Bar, 0.05 represents the nucleotide substitutions per position.

Supplementary Figure 3 Polar lipid profile of strain H1-1-2A^T^. PE, phosphatidylethanolamine; DPG, diphosphatidylglycerol; PG, phosphatidylglycerol; APL, aminophospholipid; PL, unidentified phospholipid; L, unidentified lipid.

Supplementary Table 1 Nitrogen fixation genes predicted from the genome of strain H1-1-2A^T^.

Supplementary Table 2 Denitrification genes predicted from the genome of strain M9-3-2^T^.

Supplementary Table 3 Sulfur oxidation genes found in the genome of strain H1-1-2A^T^.

Supplementary Table 4 Genomic features of the members affiliated to the family *Zoogloeaceae*.
