# Supplementary figures and images for: Nitrogeniibacter aestuarii sp. nov., a Novel Nitrogen-Fixing Bacterium Affiliated to the Family Zoogloeaceae and Phylogeny of the Family Zoogloeaceae Revisited
Source: Front Microbiol. 2021 Oct 20;12:755908. doi: 10.3389/fmicb.2021.755908 (PMC8565577; doi:10.3389/fmicb.2021.755908)

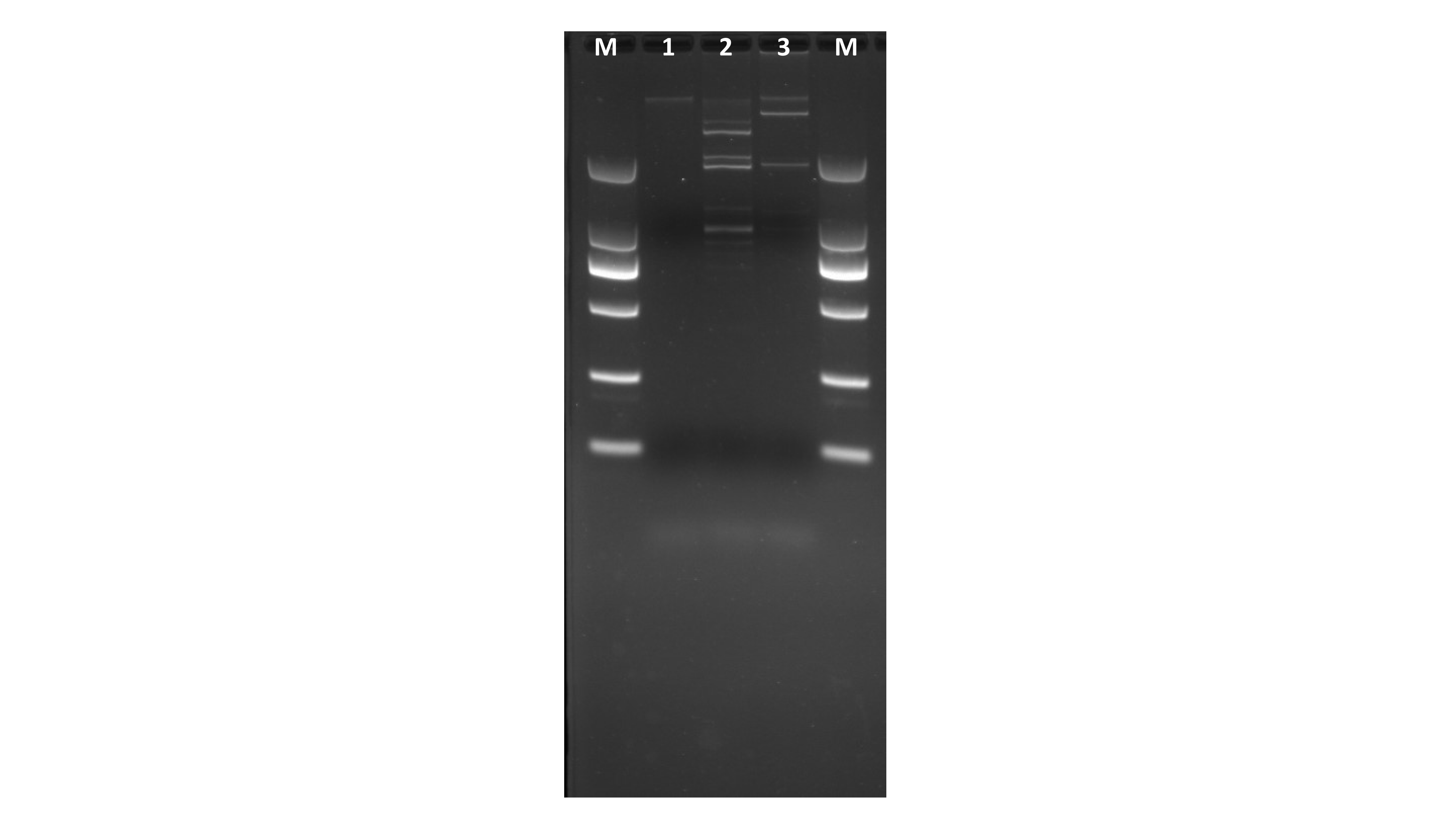

Supplement: Supplementary Figure 1 — Electrophoresis pattern of BOX-PCR fingerprinting of the strains. M. DL 2000 DNA marker; 1, strain M9-3-2T; 2, strain H1-1-2AT; 3, strain ZN11-R3-1. [file Image_1.TIF]

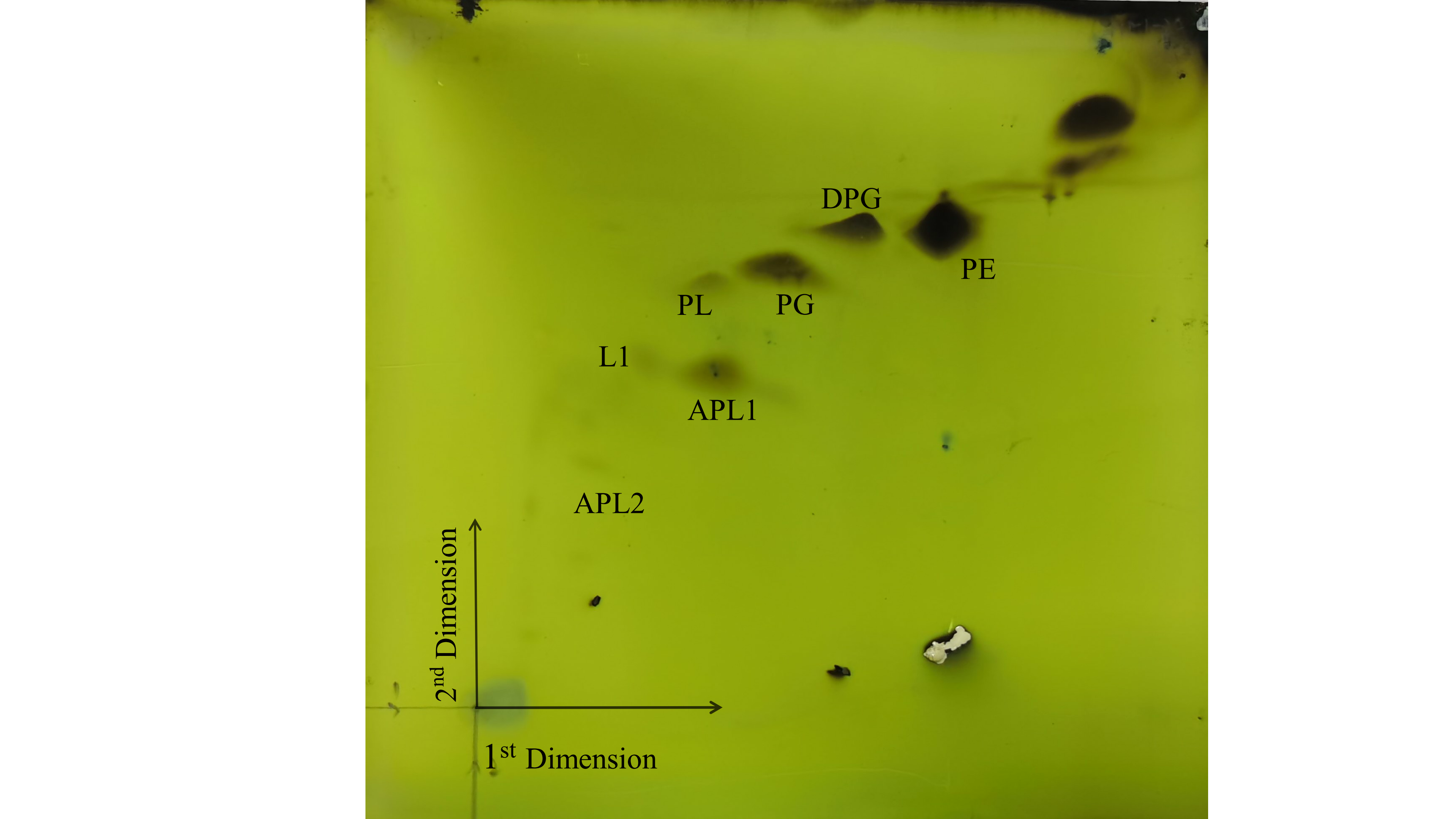

Supplement: Supplementary Figure 3 — Polar lipid profile of strain H1-1-2AT. PE, phosphatidylethanolamine; DPG, diphosphatidylglycerol; PG, phosphatidylglycerol; APL, aminophospholipid; PL, unidentified phospholipid; L, unidentified lipid. [file Image_3.TIF]
