# Supplementary material for: Nitrogeniibacter aestuarii sp. nov., a Novel Nitrogen-Fixing Bacterium Affiliated to the Family Zoogloeaceae and Phylogeny of the Family Zoogloeaceae Revisited
Source: Front Microbiol. 2021 Oct 20;12:755908. doi: 10.3389/fmicb.2021.755908 (PMC8565577; doi:10.3389/fmicb.2021.755908)
Supplement: Supplementary file 8 [file Table_5.DOCX]

**Table S5**. Comparison of chemotaxonomic characteristics among the closely related genera.

| Characteristics | *Nitrogeniibacter* | *Cognatazoarcus*^a^ | *Pseudazoarcus*^b^ | *Pseudothauera*^c^ | *Parazoarcus*^d^ | *Thauera^e^* | *Azoarcus^f^* |
| --- | --- | --- | --- | --- | --- | --- | --- |
| Type species | *Nitrogeniibacter mangrovi* | ‘*Azoarcus halotolerans*’ | ‘*Azoarcus pumilus*’ | ‘*Thauera hydrothermalis*’ | ‘*Azoarcus communis*’ | *Thauera selenatis* | *Azoarcus indigens* |
| Quinone | Q-8 | Q-8 | Q-8 and Q-7 | Q-8 | ND | Q-8 | Q-8 |
| Major fatty acids | C_16:0_, SF3 | C_16:0_, SF3 | C_16:0_, SF3 | C_16:0_, SF3 | C_16:0_, SF3 | C_16:0_, C_16:1_ *ω*7*c*, C_18:1_ *ω*7*c* | C_16:0_, SF3 |
| Major lipids | DPG, PE, PG, PL, APL | DPG, PE, PG, PL, GPL | DPG, PE, PG, GPL, GL, APL | DPG, PE, PG, PL, AL | DPG, PE, PG, PL, APL^f^ | DPG, PE, PG, PL, L | DPG, PE, APL, PL |
| Genome size (Mbp) | 4.24-4.75 | 4.97 | 3.22-4.22 | 3.06-4.72 | 5.0 | 3.69-5.29 | 4.45-5.46 |
| DNA G+C content (%) | 62.7-67.1 | 63.5 | 62.8-66.5 | 63.6-68.3 | 62.5 | 64.3-68.6 | 67.6-67.8 |

1. Data was taken from ([Li et al., 2020](#_ENREF_3)).
2. Data was taken from ([Fu et al., 2019](#_ENREF_2)).
3. Data was taken from ([Yang et al., 2018](#_ENREF_7)).
4. Data was taken from ([Reinhold-Hurek et al., 1993](#_ENREF_6); [Chen et al., 2013](#_ENREF_1)).
5. Data was taken from ([Macy et al., 1993](#_ENREF_4); [Qiao et al., 2018](#_ENREF_5)).
6. Data was taken from ([Chen et al., 2013](#_ENREF_1)).

ND, no data; SF3, summed feature 3, comprised C_16:1_ *ω*7*c* and/or C_16:1_ *ω*6*c*. DPG, diphosphatidylglycerol; PE, phosphatidylethanolamine; PG, phosphatidylglycerol; GPL, unidentified phosphoglycolipids; APL, unidentified aminophospholipids; AL, unidentified aminolipids; L, unidentified lipids.

Reference

Chen, M.H., Sheu, S.Y., James, E.K., Young, C.C., and Chen, W.M. (2013). *Azoarcus olearius* sp. nov., a nitrogen-fixing bacterium isolated from oil-contaminated soil. *Int J Syst Evol Microbiol* 63(Pt 10)**,** 3755-3761. doi: 10.1099/ijs.0.050609-0.

Fu, G.Y., Yu, X.Y., Yu, X.D., Zhao, Z., Chen, C., Wang, R.J., et al. (2019). *Azoarcus pumilus* sp. nov., isolated from seawater in Sanya, China. *Int J Syst Evol Microbiol* 69(5)**,** 1459-1464. doi: 10.1099/ijsem.0.003341.

Li, S., Zhao, L., Han, J., Liu, S., Dai, J., Fu, G., et al. (2020). *Azoarcus halotolerans* sp. nov., a novel member of *Rhodocyclaceae* isolated from activated sludge collected in Hong Kong. *Int J Syst Evol Microbiol* 70(11)**,** 5799-5805. doi: 10.1099/ijsem.0.004476.

Macy, J.M., Rech, S., Auling, G., Dorsch, M., Stackebrandt, E., and Sly, L.I. (1993). *Thauera selenatis* gen. nov., sp. nov., a member of the beta subclass of *Proteobacteria* with a novel type of anaerobic respiration. *Int J Syst Bacteriol* 43(1)**,** 135-142. doi: 10.1099/00207713-43-1-135.

Qiao, N., Xi, L., Zhang, J., Liu, D., Ge, B., and Liu, J. (2018). Thauera sinica sp. nov., a phenol derivative-degrading bacterium isolated from activated sludge. *Antonie Van Leeuwenhoek* 111(6)**,** 945-954. doi: 10.1007/s10482-017-0993-5.

Reinhold-Hurek, B., Hurek, T., Gillis, M., Hoste, B., Vancanneyt, M., Kersters, K., et al. (1993). *Azoarcus* gen. nov., nitrogen-fixing *Proteobacteria* associated with roots of Kallar grass (*Leptochloa fusca* (L.) Kunth), and description of two species, *Azoarcus indigens* sp. nov. and *Azoarcus communis* sp. nov. *Int J Syst Bacteriol* 43(3)**,** 574-584. doi: doi.org/10.1099/00207713-43-3-574.

Yang, L., Muhadesi, J.B., Wang, M.M., Wang, B.J., Liu, S.J., and Jiang, C.Y. (2018). *Thauera hydrothermalis* sp. nov., a thermophilic bacterium isolated from hot spring. *Int J Syst Evol Microbiol* 68(10)**,** 3163-3168. doi: 10.1099/ijsem.0.002960.
