# Supplementary material for: Nitrogeniibacter aestuarii sp. nov., a Novel Nitrogen-Fixing Bacterium Affiliated to the Family Zoogloeaceae and Phylogeny of the Family Zoogloeaceae Revisited
Source: Front Microbiol. 2021 Oct 20;12:755908. doi: 10.3389/fmicb.2021.755908 (PMC8565577; doi:10.3389/fmicb.2021.755908)
Supplement: Supplementary file 9 [file Table_6.DOCX]

***Nitrogeniibacter aestuarii* sp. nov., a novel nitrogen-fixing bacterium affiliated to the family *Zoogloeaceae* and phylogeny of the family *Zoogloeaceae* revisited**

Zhaobin Huang^1,2^*, Renju Liu^3^, Fenghua Chen^1^, Qiliang Lai^3^, Aharon Oren^4^, and Zongze Shao^3*^

^1^College of Oceanology and Food Science, Quanzhou Normal University, Quanzhou, PR China

^2^Key Laboratory of Inshore Resources Biotechnology (Quanzhou Normal University) Fujian Province University, Quanzhou, PR China

^3^Key Laboratory of Marine Genetic Resources, Third Institute of Oceanography, Ministry of Natural Resources, Xiamen, PR China

4. The Institute of Life Sciences, The Edmond J. Safra Campus, The Hebrew University of Jerusalem, Jerusalem, Israel

*Corresponding authors.

Zhaobin Huang

E-mail: [zbhuangemail@gmail.com](mailto:zbhuangemail@gmail.com)

Zongze Shao

E-mail: [shaozz@163.com](mailto:shaozz@163.com)

Running title: *Nitrogeniibacter aestuarii* sp. nov.

**Abstract**

Members of the family *Zoogloeaceae* within the order *Rhodocyclales* are found to play vital roles in terrestrial and aquatic ecosystems by participating in biofloc formation in activated sludge, polycyclic aromatic hydrocarbon degradation, as well as nitrogen metabolism, such as denitrification and nitrogen fixation. Here, two bacterial strains designated H1-1-2A^T^ and ZN11-R3-1 affiliated to the family *Zoogloeaceae* were isolated from coastal wetland habitats. The 16S rRNA gene sequences of the two strains were 100% identical and had maximum similarity with *Nitrogeniibacter* *mangrovi* M9-3-2^T^ of 98.4%, and ≤ 94.5% with other species. Phylogenetic analysis suggested that the two strains belonged to a single species and formed a novel monophyletic branch affiliated to the genus *Nitrogeniibacter*. The average nucleotide identity (ANI) value and digital DNA-DNA hybridization (dDDH) estimate between the two strains and *N.* *mangrovi* M9-3-2^T^ were 78.5-78.7% and 21.4-21.6%, respectively, indicating that the two strains represent a novel species. The genomes of strain H1-1-2A^T^ (complete genome) and ZN11-R3-1 (draft genome) were 4.7 Mbp in length encoding ~4,360 functional genes. The DNA G+C content was 62.7%. Nitrogen ﬁxation genes were found in the two strains, which were responsible for the growth on nitrogen-free medium, whereas denitrification genes found in *N.* *mangrovi* M9-3-2^T^ were absent in the two strains. The respiratory quinone was ubiquinone-8. The major polar lipids consisted of phosphatidylethanolamine, diphosphatidylglycerol, phosphatidylglycerol and aminophospholipid. The major fatty acids were summed feature 3 (C_16:1_*ω*7*c* and C_16:1_*ω*6*c*), C_16:0_, C_12:0_, and C_10:0_ 3-OH. Based on genomic, phenotypic, and chemotaxonomic characterizations, strains H1-1-2A^T^ and ZN11-R3-1 represent a novel species of the genus *Nitrogeniibacter*, for which the name *Nitrogeniibacter aestuarii* sp. nov. is proposed. The type strain is H1-1-2A^T^ (= MCCC 1K04284^T^ = KCTC 82672^T^) and additional strain is ZN11-R3-1 (=MCCC 1A17971=KCTC 82671). Additionally, phylogenomic analysis of the members of the family *Zoogloeaceae* including type strains and uncultivated bacteria was performed, using the Genome Taxonomic Database toolkit (GTDB-Tk). Combined with the 16S rRNA gene phylogeny, four novel genera, *Parazoarcus* gen. nov., *Pseudazoarcus* gen. nov., *Pseudothauera* gen. nov., and *Cognatazoarcus* gen. nov. were proposed. This study provided new insights to the taxonomy of the family *Zoogloeaceae*.

**Keywords**: *Nitrogeniibacter*; *Zoogloeaceae*; nitrogen fixation; Polyphasic taxonomy; Phylogenomic tree

**Significance**

A novel species named *Nitrogeniibacter aestuarii* with two strains affiliated to the family *Zoogloeaceae* was proposed by using a polyphasic taxonomic approach. The species had the ability of nitrogen fixation, which was assumed to play important roles in the nitrogen cycle of coastal wetlands. Additionally, phylogenetic analysis of the family *Zoogloeaceae* based on genome sequences of type strains and uncultivated bacteria was performed and four novel genera, *Parazoarcus* gen. nov., *Pseudothauera* gen. nov., *Pseudazoarcus* gen. nov., and *Cognatazoarcus* gen. nov. were proposed. This study provided new insights into the taxonomy of the family *Zoogloeaceae.*

**Introduction**

The family *Zoogloeaceae* as a member of the order *Rhodocyclales* was firstly proposed in 2017 ([Boden et al., 2017](#_ENREF_2)). Thus far, six genera with validly published names were described (<https://lpsn.dsmz.de/family/zoogloeaceae>), including *Zoogloea* ([Shin et al., 1993](#_ENREF_35)), *Azoarcus* ([Rabus et al., 2019](#_ENREF_31)), *Aromatoleum* ([Rabus et al., 2019](#_ENREF_31)), *Thauera* ([Macy et al., 1993](#_ENREF_25)), *Uliginosibacterium* ([Weon et al., 2008](#_ENREF_38)), and *Nitrogeniibacter* ([Liao et al., 2021](#_ENREF_21)). Members of this family were found to play vital roles in terrestrial and aquatic habitats by participating in biofloc formation in activated sludge (such as *Zoogloea*) ([Shin et al., 1993](#_ENREF_35)), polycyclic aromatic hydrocarbon (PAH) degradation (*Thauera*) ([Mechichi et al., 2002](#_ENREF_26)), as well as nitrogen metabolism, such as denitrification (*Thauera* and *Nitrogeniibacter*) ([Liu et al., 2013](#_ENREF_23); [Liao et al., 2021](#_ENREF_21)) and nitrogen fixation (*Azoarcus*) ([Lin et al., 2020](#_ENREF_22)).

Previously circumscription of the taxonomy of the family *Zoogloeaceae* depended largely on phylogeny of 16S rRNA gene sequences and a small number of species were included ([Boden et al., 2017](#_ENREF_2)). The family *Zoogloeaceae* currently includes nearly 50 species with validly published or effectively published names (<https://www.ezbiocloud.net/taxonomy?tn=Zoogloeaceae>). With the advance of next-generation sequencing (NGS) and methods of constructing metagenomic-centric genomes and single-cell genomes used for uncultivated bacteria ([Rinke et al., 2013](#_ENREF_34); [Parks et al., 2018](#_ENREF_28); [Lapidus and Korobeynikov, 2021](#_ENREF_17)), a large number of genomes affiliated to the family *Zoogloeaceae* and the order *Rhodocyclales* were obtained and released publically in the Genome portal of GenBank. These genomes were obtained from various habitats including wastewater, soil, sediment and freshwater ([Wang et al., 2020](#_ENREF_37)). The genomes of uncultivated *Zoogloeaceae* members expanded our knowledge on their ecological niches and phylogenetic diversity. However, the taxonomic position of several members of the family *Zoogloeaceae* is still controversial. For instance, the genus *Niveibacterium* proposed in the family *Rhodocyclaceae* ([Chun et al., 2016](#_ENREF_6)) is placed as a member within the *Zoogloeaceae* in the EzBioCloud Database ([Yoon et al., 2017a](#_ENREF_40)); *Thauera hydrothermalis* GD-2^T^ formed a separate branch on the basis of phylogeny of 16S rRNA gene that were distinct from the type species *T. selenatis* ATCC 55363^T^ ([Liao et al., 2021](#_ENREF_21)). This may be the result of using a small number of species for phylogenetic analysis based on 16S rRNA gene comparison. Thus, the phylogenetic relationship of the *Zoogloeaceae* members needs to be reconsidered, especially on the basis of genome sequences. The Genome Taxonomic Database (GTDB) is considered to be a reliable tool to deﬁne the bacterial taxonomic ranks using 120 conserved concatenated proteins ([Parks et al., 2018](#_ENREF_28)) and is used in accurate assignment for not only the described species but also for genomes of uncultivated organisms. Thus, the phylogeny of the family *Zoogloeaceae* was revisited in this study based on the use of GTDB tools.

*Nitrogeniibacter*, affiliated to the family *Zoogloeaceae*, is a recently proposed genus, with a single species, *N. mangrovi*. The type strain M9-3-2^T^ (=MCCC 1K03313^T^ =JCM 32045^T^) was isolated from an enrichment culture of mangrove sediment ([Liao et al., 2021](#_ENREF_21)). The genus is circumscribed on the basis of 16S rRNA gene phylogeny and concatenated core genes (phylogenomic tree) and physiological and chemical characteristics ([Liao et al., 2021](#_ENREF_21)). The cells are Gram-stain-negative, show anaerobic and aerobic growth, rod-shaped, oxidase-positive, and catalase-positive. Ubiquinone-8 (Q-8) is the major respiratory quinone, diphosphatidylglycerol, phosphatidylethanolamine, phosphatidylglycerol, phospholipids, and aminophospholipids are major polar lipids, and summed feature 3 (C_16:1_*ω*7*c* and C_16:1_*ω*6*c*), C_16:0_, C_10:0_ 3-OH, C_14:0_ and C_10:0_ are major fatty acids. This genus had the ability of denitrification under both aerobic and anaerobic conditions.

In this study, two isolates designated H1-1-2A^T^ and ZN11-R3-1 were obtained from a sediment sample of a *Spartina alterniflora* wetland and from styrofoam plastics collected from a mangrove, respectively. The isolates were found to have identical 16S rRNA gene sequences and likely represented a novel species of the genus *Nitrogeniibacter* within the family *Zoogloeaceae*. This study aimed to determine the taxonomic status of the two isolates using a polyphasic taxonomic approach. Additionally, the phylogeny of the *Zoogloeaceae* members was elucidated based on the available genomes to further advance the taxonomy of the family.

**Materials and methods**

**Bacterial isolation and cultivation**

Strains H1-1-2A^T^ and ZN11-R3-1 were isolated from a coastal sediment sample and from an enrichment culture inoculated with coastal styrofoam plastics, respectively. The sediment sample was collected from a *Spartina alterniflora* growing area in a wetland (24°86' N, 118°68' E) in Quanzhou Bay, Quanzhou, PR China on Sep. 05, 2019. A water-extracted medium (WEM) prepared using the nutrients extracted from the sediment with pure water (w/v=1:1) was used to isolate strain H1-1-2A^T^ ([Huang et al., 2020b](#_ENREF_13)). The 0.2 g sediment sample was subjected to 10-fold serial dilutions, and spread on the WEM plates and incubated for 2 weeks at 28^o^C. Strain H1-1-2A^T^ was picked and then streaked onto Marine Broth 2216 (MB, BD) agar plates to obtain a pure culture. For the isolation of strain ZN11-R3-1, styrofoam plastic was collected from a mangrove preservation area (24^o^27' N, 117^o^53' E) in Longhai, Zhangzhou, PR China on Nov. 23, 2019. The plastics were placed into an enrichment medium of 300 ml sterile MMC (NaCl 24 g/L; MgSO_4_·7H_2_O 7.0 g/L; NH_4_NO_3_ 1 g/L; KCl 0.7 g/L; KH_2_PO_4_ 2.0 g/L and Na_2_HPO_4_·12H_2_O 3.0 g/L, pH=7.4), and maintained at 150 rpm shaking at 28^o^C for 2 months. An aliquot (2 ml) of enriched culture was then transferred to another 100 ml fresh MMC medium containing sterile plastics, and cultured for another 2 months. Then the enrichment was repeated as above. The biomass in the third enrichment culture was collected using centrifugation at 6,000 rpm for 15 min, and plated on an MB agar plate and maintained at 30^o^C. Strains H1-1-2A^T^ and ZN11-R3-1 grew well on MB agar plates and MB medium, and were stored at -80 ^o^C with 20% glycerol (v/v) in the laboratory.

**Phylogeny analysis based on 16S rRNA gene sequences**

The nearly-complete 16S rRNA gene sequences of strain H1-1-2A^T^ and strain ZN11-R3-1 were obtained using Sanger sequencing performed as described in a previous study ([Huang et al., 2020b](#_ENREF_13)). The sequences were also compared with rRNA genes extracted from the genome sequences.

Sequences of the closely related relatives of the two strains were obtained from the EzBioCloud database ([Yoon et al., 2017a](#_ENREF_40)) and the NCBI nucleotide database (<https://blast.ncbi.nlm.nih.gov/Blast.cgi>). *Burkholderia cepacia* ATCC 25416^T^ was selected as an outgroup. Then, the 16S rRNA gene sequences were aligned and subjected to phylogenetic analysis using two algorithms, neighbor-joining (NJ) and maximum likelihood (ML) methods with 1,000 bootstraps using MEGA 7.0 ([Huang et al., 2019](#_ENREF_12)). The best model (T92+G+I) with the lowest Bayesian Information Criterion (BIC) scores was selected.

**Genome sequencing and gene annotation**

The draft genome sequences of strain H1-1-2A^T^ and strain ZN11-R3-1 were determined using the Illumina HiSeq X-Ten platform (Shanghai Majorbio Bio-Pharm Technology Co., Ltd, Shanghai, China). A library of ~400 bp fragments was constructed and paired-end (PE) short reads of ~ 1 Gb were obtained. The PE reads were firstly trimmed to remove the low base of quality <20 and length <50 bp using sickle (<https://github.com/najoshi/sickle>). Then, clean reads were assembled into contigs using SPAdes v 3.8.0 with a serial of *k* values of 21, 33, 55, 77, 99, 127 and –*careful* flag ([Huang et al., 2019](#_ENREF_12)). Then, contigs shorter than 1 kb were removed from the assembled contigs. The genome quality was evaluated using QUAST ([Gurevich et al., 2013](#_ENREF_9)).

The complete genome of type strain H1-1-2A^T^ was obtained using PacBio sequencing with one SMART cell. The 10-kb fragment library was constructed followed the manufacturer’s instructions. The long reads were assembled using the SMRT Link (V6.0.0.47841) of PacBio.

The complete 16S rRNA gene sequence was extracted from the genome sequence using RNAmmer ([Lagesen et al., 2007](#_ENREF_16)). Genome completeness was evaluated using CheckM v1.0.1 ([Parks et al., 2015](#_ENREF_29)). Gene annotation was carried out using the RAST server ([Aziz et al., 2008](#_ENREF_1)) and the KAAS system (https://www.genome.jp/tools/kaas/). Functional genes with high similarity to close relatives were searched using the blast+ program with e-value cutoff of 1e-5 ([Camacho et al., 2009](#_ENREF_4)).

The average nucleotide identity (ANI) values were estimated using OrthoANI computation on the EzBioCloud Database ([Yoon et al., 2017b](#_ENREF_41)). Digital DNA-DNA Hybridization (dDDH) estimates were calculated on the GGDC website (<https://ggdc.dsmz.de/>). Average amino acids identity among genomes was calculated using CompareM v0.1.2 (<https://github.com/dparks1134/CompareM>). The percentage of conserved proteins (POCP), proposed as genus boundary values was also calculated for genomic comparison ([Qin et al., 2014](#_ENREF_30)).

**Phylogenomic analysis**

The genomes affiliated to the order *Rhodocyclales* were downloaded from the genome portal in NCBI (<https://www.ncbi.nlm.nih.gov/genome/>). A total of 303 genomes were obtained (until Feb.19, 2021) and the genome quality was checked using CheckM v1.0.1 ([Parks et al., 2015](#_ENREF_29)). Genomes of <50% completeness and >10% contamination were removed from the following study. In addition, 9 genomes, identified using GTDB-tk v. 0.3.2 ([Chaumeil et al., 2019](#_ENREF_5)), did not belong to the order *Rhodocyclales*, and these were removed from the study. Then, the phylogenomic tree of the genomes was inferred using a concatenated alignment of 120 bacterial single-copy genes with GTDB-tk v. 0.3.2 by using FastTree ([Parks et al., 2018](#_ENREF_28)). The tree was edited using the Interactive Tree of Life (iTOL) online ([Letunic and Bork, 2007](#_ENREF_19)). In addition, a phylogenomic tree based on the genomes of type strains belonging to the order *Rhodocyclales* was also constructed using GTDB-tk.

**Phenotypic properties**

Gram staining was carried out using a Gram staining kit (Hangzhou Tianhe Microorganism Reagent, Co. Ltd.). Colony morphology was recorded on a MB agar plate after incubation at 30 ^o^C for 3 days. Catalase activity was tested by using 3% H_2_O_2_ solution. Oxidase activity was tested using the oxidase reagent (1% aqueous solution of N,N,N',N'-tetramethyl-p-phenylenediamine dihydrochloride, bioMérieux, France). Motility was observed by puncturing the cells into 0.5% agar. Growth under the anaerobic condition was tested by inoculating the cells into an anaerobic MB medium for 7 days. The growth temperature range, NaCl tolerance range, pH range of the strains, and hydrolysis of substrates were determined as described in our previous study ([Huang et al., 2020b](#_ENREF_13)). Growth on nitrogen-free medium was tested following the method of Huang et al. with 5 g/L NaCl and 5 g/L glucose ([Huang et al., 2014](#_ENREF_10)). *N. mangrovi* M9-3-2^T^ (=MCCC 1K03313^T^), obtained from the Marine Culture Collection Center (MCCC), was used as a reference strain.

Physiological and biochemical characterization was carried out using API ZYM, API 20NE, and API 20E kits according to the manufacturer’s instructions (bioMérieux, France). The tested strains and the reference strain were maintained under identical laboratory conditions. Test strips were maintained at 35^o^C for determining the physiological and biochemical properties.

**Chemotaxonomic characteristics**

For the analysis of fatty acids composition, the strains and reference strain were cultured in MB at 35 ^o^C for 3 days and cells were collected by centrifugation at 8,000 rpm for 10 min. The cellular fatty acids were saponified, methylated and extracted, and then identified following the standard MIDI protocol (Sherlock Microbial Identification System, version 6B).

For the polar lipids analysis, strain H1-1-2A^T^ was cultured in MB medium at 35 ^o^C for 3 days, and cells were harvested by using centrifugation as above. Polar lipids were extracted using a chloroform/methanol system and analysed using one- and two-dimensional TLC using Merck silica gel 60 F254 aluminum-backed thin-layer plates. Lipids were detected and identified by spraying the specific reagents ([Huang et al., 2020a](#_ENREF_11)).

**Results and Discussion**

**Phylogeny of 16S rRNA gene sequences**

The 16S rRNA gene sequences of strains H1-1-2A^T^ and ZN11-R3-1, obtained by Sanger sequencing or extracted from the genome sequences, had 100% identity, indicating the two strains belonged to same species. The BOX-PCR genotypic fingerprinting profiles of two strains were similar but distinctive (**Supplementary Figure 1**), which confirmed that they were not clonal. Also, the fingerprinting of the two strains were totally different from *N. mangrovi* M9-3-2^T^, indicating they may belong to a novel species different from *N. mangrovi.*

Sequence similarity search showed that the 16S rRNA gene sequence of strain H1-1-2A^T^ had the maximum similarity (99.6%) with an uncultured bacterium clone IWNB003 (accession number: FR744543), followed by *N. mangrovi* M9-3-2^T^ (98.4%), and had sequence similarities of ≤ 94.5% with other species affiliated to the family *Zoogloeaceae*. The clone IWNB003 was found in nitrate-amended injection seawater from an oil field ([Gittel et al., 2012](#_ENREF_8)), and *N. mangrovi* M9-3-2^T^ has the ability of denitrification ([Liao et al., 2021](#_ENREF_21)), which may indicate that *Nitrogeniibacter* members play valuable roles in nitrogen cycle in the environment.

Phylogeny of 16S rRNA gene sequence inferred from the ML and NJ methods placed strains H1-1-2A^T^ and ZN11-R3-1 within the genus *Nitrogeniibacter* as a novel monophyletic line, distinct from *N. mangrovi* M9-3-2^T^. This indicated that the two strains could be considered as a novel species of the genus *Nitrogeniibacter* (**Figure 1 and Supplementary Figure 2**).

Phylogeny of 16S rRNA gene sequences indicated that the members of *Azoarcus* as well as the members of *Thauera* were separated into different clades, which were clearly separated from the type species, *A. indigens* and *T. selenatis*. Firstly, *A. pumilus* SY39^T^ and ‘*A*. *taiwanensis*’ NSC3^T^ formed a separate cluster, which did not cluster with the type species *A. indigens*. Here, we named this cluster as a novel genus *Pseudazoarcus*, which was equal to the group name ‘Azoarcus_D’ of the Genome Taxonomy Database (GTDB) ([Chaumeil et al., 2019](#_ENREF_5)). Thus, *A. pumilus* should be transferred into the genus *Pseudazoarcus*. *Azoarcus pumilus* was designated the type species of this genus and was renamed as *Pseudazoarcus pumilus* comb. nov. ‘*A*. *taiwanensis*’ (a name effectively but not validly published) ([Lee et al., 2014](#_ENREF_18)), was also affiliated to this genus. Secondly, in the phylogenetic clade of the genus *Thauera*, there were four species, including *T. lacus*, *T. hydrothermalis*, *A. nasutitermitis* and *A. rhizosphaerae* that formed a monophyletic cluster. Though this cluster formed a node with other *Thauera* members, bootstrap support was low (<70% of both ML and NJ) (**Figure 1 and Supplementary Figure 2**). The four species may be assigned to a new genus named *Pseudothauera*, which is equivalent to ‘Thauera_A’ in the GTDB taxonomy. Thus, the four species, *T. lacus*, *T. hydrothermalis*, *A. nasutitermitis* and *A. rhizosphaerae* should be transferred to a novel genus *Pseudothauera* and renamed as *Pseudothauera lacus* comb. nov., *Pseudothauera hydrothermalis* comb. nov., *Pseudothauera nasutitermitis* comb. nov. and *Pseudothauera rhizosphaerae* comb. nov., respectively. Thirdly, *A*. *halotolerans* HKLI-1^T^ formed an independent line on the phylogenomic tree, which clearly branched with *Azoarcus*. This species should be reclassified into a novel genus; *Cognatazoarcus halotolerans* gen. nov., comb. nov. was therefore proposed. Fourthly, *A. communis* SWub3^T^ did not cluster together with the type species *A. indigens*, and should be transferred into a novel genus. Here, we named this cluster as a novel genus *Parazoarcus*, which was equal to the genus name ‘Azoarcus_C’ of the Genome Taxonomy Database (GTDB).

**Genomic characteristics**

The complete genome of strain H1-1-2A^T^ included one chromosome (4,678,511 bp) and one plasmid (66,515 bp). The draft genome size of strain ZN11-R3-2 was 4,656,485 bp on 42 contigs (>1 kb) with N50 value of 343,918 bp (**Table 1**). Three copies of *rrn* operon (16S-

23S-5S rRNA genes) were found in the complete genome, and 16S rRNA gene copies were 1,528 bp in length and had 100% identity. The genome size of the strains was a little larger than *N. mangrovi* M9-3-2^T^ (a chromosome of 4,236,644 bp; accession number: CP048836). The DNA G+C content of strains H1-1-2A^T^ and ZN11-R3-1 were 62.67% and 62.71%, respectively, which were a little lower than *N. mangrovi* M9-3-2^T^ (67.13%). Gene prediction showed that there were 4,364 and 3,884 predicted genes in strains H1-1-2A^T^ and M9-3-2^T^, respectively. dDDH and ANI values between strain H1-1-2A^T^ and ZN11-R3-1 were estimated to be 85.6% and 98.4%, respectively. These values exceeded the threshold of species delineation, which strongly supported that the two strains belonged to the same species ([Kim et al., 2014](#_ENREF_14)). dDDH and ANI values between strains H1-1-2A^T^ and ZN11-R3-1 and *N. mangrovi* M9-3-2^T^ were 21.4-21.6% and 78.50-78.72%, indicating that strains H1-1-2A^T^ and ZN11-R3-1 represented a novel species.

Strains H1-1-2A^T^ and ZN11-R3-1 contained nitrogen fixation gene clusters encoding nitrogenase reductase (*nifH*, KO list: K02588), nitrogenase molybdenum-iron protein (*nifD*, K02586; *nifK*, K02591), and related proteins (*modABCD*) (**Supplementary Table 1**), which were also found in *N. mangrovi* M9-3-2^T^ ([Liao et al., 2021](#_ENREF_21)). The nitrogen fixation genes were assumed to enable their growth on nitrogen-free medium. Interestingly, denitrification genes, *nirBD* (K000362 and K000363), *norBC* (K004561 and K002305*)*, and *narGHIJ* (K000370, K000371, K000373, and K000374) were not found in strains H1-1-2A^T^ and ZN11-R3-1, but they were present in *N. mangrovi* M9-3-2^T^ (**Supplementary Table 2**), in which denitrification was confirmed in a laboratory experiment ([Liao et al., 2021](#_ENREF_21)). In addition, the *sox* system (*soxABCDXYZ* gene cluster) was present in strain H1-1-2A^T^ (**Supplementary Table 3**) and in *N. mangrovi* M9-3-2^T^ ([Liao et al., 2021](#_ENREF_21)), suggesting that *Nitrogeniibacter* members may have the ability of sulfur oxidation.

**Phylogenomics of the family *Zoogloeaceae***

The development of MAG binning and single cell genomes contributed large numbers of genome sequences of uncultivated bacteria, including members of family *Zoogloeaceae* and the order *Rhodocyclales*, which could expand knowledge on the phylogenetic diversity based on core genome analysis.  Here, the genomes of the order *Rhodocyclales* with ≥50% completeness and ≤10% contamination were used, of which the genome quality was verified to perform accurate phylogenetic analysis by GTDB-tk ([Bowers et al., 2017](#_ENREF_3)). A total of 277 genomes affiliated to the order *Rhodocyclales* that meet the above standards were used in the phylogenomic analysis. Compared to the 78 and 92 genomes analysed in phylogenomic studies of the order *Rhodocyclales* by ([Wang et al., 2020](#_ENREF_37)) and ([Liao et al., 2021](#_ENREF_21)), respectively, our study further expanded the known phylogenetic groups within the order *Rhodocyclales*. The described species account for a minor part of the phylogenomic tree, indicating that majority of the members of *Rhodocyclales* are still waiting to be cultivated (**Figure 2**).

Phylogenomic analysis based on 120 bacterial conserved single-copy genes strongly placed strains H1-1-2A^T^ and ZN11-R3-1 in a sister group of the genus *Nitrogeniibacter*, which was neighbored by ‘*Denitromonas*’. This agreed with the phylogeny based on concatenated core-genome sequences ([Liao et al., 2021](#_ENREF_21)). ‘*Denitromonas*’ should be transferred into the family *Zoogloeaceae*, and did not belong to the family *Rhodocyclaceae* (<https://lpsn.dsmz.de/family/rhodocyclaceae>). In the lineages of the family *Zoogloeaceae*, the relationship between *A. pumilus* SY39^T^ and ‘*A*. *taiwanensis*’ NSC3^T^ showed congruent topology with 16S rRNA gene phylogeny (**Figure 1**), which strongly supported the two species should be reclassified into a novel genus, for which we propose the name *Pseudazoarcus*. Also, in the phylogenomic tree, *T. lacus* D20^T^, *T. hydrothermalis* GD-2^T^, *A. nasutitermitis* CC-YHH838^T^ and *A. rhizosphaerae* CC-YHH848^T^ formed a monophyletic cluster, which also supported the phylogeny of the 16S rRNA gene. The four species should be assigned to a new genus, for which we propose the name *Pseudothauera*. In addition, phylogenomic analysis of *A. olearius* DQS-4^T^, *A. indigens* VB32^T^ and *A. communis* SWub3^T^ showed topology incongruent with the 16S rRNA gene, possibly due to the small number of sequences used. Thus, it is proposed that *A. communis* SWub3^T^ be reclassified into a novel genus named *Parazoarcus* gen. nov. *Azoarcus halotolerans* HKLI-1^T^, which is only distantly related to the type species *A. indigens*, should also be reclassified into a novel genus. Thus, *Cognatazoarcus* gen. nov. was proposed. *Niveibacterium* firstly proposed in the family *Rhodocyclaceae* ([Chun et al., 2016](#_ENREF_6)) should be transferred to the family *Zoogloeaceae* based on the phylogenetic analysis. Finally, a family-level lineage including the genus *Rugosibacter* was clearly separated from the family *Zoogloeaceae*, indicating that *Rugosibacter* may represent a novel family.

**Figure 3** presents a small phylogenomic tree reconstructed using GTDB-tk, only including the type strains. Two genomes, *Thauera selenatis* AX^T^ (type species) with high genome contamination and ‘*Zoogloea ramigera*’ ATCC 19544, possibly incorrectly named, were excluded (**Supplementary Table 4**). The topology of the small tree was congruent with that of the large tree, which supported the above analysis. AAI values calculated among the 40 members of the family *Zoogloeaceae* ranged from 60.34% to 94.53% (**Figure 4**), which exceeded the family boundary of >45% ([Konstantinidis et al., 2017](#_ENREF_15)). Thus, the members should be considered to belong to the family *Zoogloeaceae*. Our analysis did not support the proposal of *Uliginosibacterium* as an independent family ([Wang et al., 2020](#_ENREF_37)). Compared to POCP values, AAI values demonstrated certain advantages to delineate the genus boundary of the members of the *Zoogloeaceae* (**Figure 4**). The calculation of POCP values depends on the similarity of the protein contents of genomes, which had similar genome size ([Qin et al., 2014](#_ENREF_30)). It is reported that POCP values is also not effective and appropriate for delineating the genera of the families *Acetobacteraceae* ([Rai et al., 2021](#_ENREF_32)), *Rhodobacteraceae* ([Suresh et al., 2019](#_ENREF_36)), and *Methylococcaceae* ([Orata et al., 2018](#_ENREF_27)). For instance, the four species, *T. lacus*, *T. hydrothermalis*, *A. nasutitermitis* and *A. rhizosphaerae* clearly grouped together, ranging from 78.37 to 80.80% of the AAI values for the type strains, which were below the recommended genus cutoff of <80% ([Luo et al., 2014](#_ENREF_24)). The four species were distinctly separated from *Thauera* members and other genera (**Figure 4**). The AAI values of *Nitrogeniibacter* compared to the genera *Thauera*, *Parazoarcus*, *Azoarcus*, *Pseudothauera*, *Pseudazoarcus*, and *Cognatazoarcus* were 65.5-67.4%, 65.9-66.8%, 65.6-67.0%, 66.2-68.5%, 64.1-66.1%, and 66.4-67.5% respectively, which were below the genus cutoff of <80% ([Luo et al., 2014](#_ENREF_24)). Thus, our study expanded the family *Zoogloeaceae* into 11 genera, including *Zoogloea*, *Azoarcus*, *Aromatoleum*, *Thauera*, *Niveibacterium*, *Uliginosibacterium*, *Nitrogeniibacter*, *Parazoarcus*, *Cognatazoarcus*, *Pseudazoarcus* and *Pseudothauera*.

**Phenotypic properties**

Colonies of strains H1-1-2A^T^ and ZN11-R3-1 on MB agar plates at 30^o^C were round, transparent, convex, and ~1 mm in diameter. The cells were rod-shaped, motile and stained Gram-negative. Catalase activity and oxidase activity were found to be positive, similar to *N. mangrovi* M9-3-2^T^. The tested strains did not degrade soluble starch, skim milk, carboxymethyl cellulose, and Tweens 40, 60, and 80. Anaerobic growth was not observed for strains H1-1-2A^T^ and ZN11-R3-1. The two strains can grow at 15-40 °C with the optimum at 35°C, and a pH range of 7.0-8.0 (**Table 1**). NaCl tolerance was observed at 0-4% (w/v) with the optimum of 0.5%, similar to *N. mangrovi* M9-3-2^T^ (**Table 1**). Strains H1-1-2A^T^ and ZN11-R3-1 can grow on nitrogen-free medium, similar to *N. mangrovi* M9-3-2^T^. Nitrate cannot be reduced by strains H1-1-2A^T^ and ZN11-R3-1, in contrast to *N. mangrovi* M9-3-2^T^ which has denitrification ability. Additional biochemical and physiological properties of strains H1-1-2A^T^ and ZN11-R3-1 are listed in the species description.

**Chemotaxonomic properties**

The respiratory quinone of strain H1-1-2A^T^ was ubiquinone-8 (Q-8), as in the related *N. mangrovi* M9-3-2^T^ and other members of family *Zoogloeaceae* ([Liao et al., 2021](#_ENREF_21)). The polar lipids consisted of phosphatidylethanolamine (PE), diphosphatidylglycerol (DPG), and phosphatidylglycerol (PG), two unidentified aminophospholipid (APL), one other phospholipid (PL), and one unidentified lipid (L) (**Supplementary** **Figure 3**). The predominant fatty acids (>5%) of strain H1-1-2A^T^ consisted of summed feature 3 (43.2%), C_16:0_ (23.0%), C_12:0_ (9.5%), and C_10:0_ 3-OH (7.6%), similar to strain ZN11-R3-1 (37.9%, 26.1%, 6.3%, and 5.1%, respectively) (**Table 2**). Although the major fatty acids of strain H1-1-2A^T^ and strain ZN11-R3-1 were similar to *N. mangrovi* M9-3-2^T^, the presence of minor fatty acids such as C_18:0_ showed characteristic diﬀerences. The major isoprenoid quinone and major fatty acids of *Nitrogeniibacter* were similar to the closely related genera, *Cognatazoarcus*, *Pseudazoarcus*, *Pseudothauera* and *Parazoarcus*, and their polar lipids composition showed somewhat different profiles (**Supplementary** **Table 5**).

**Conclusion**

Based on the genomic, phylogenetic, phenotypic, and chemotaxonomic characteristics, strains H1-1-2A^T^ and ZN11-R3-1 represent a novel species of the genus *Nitrogeniibacter*, for which the name *Nitrogeniibacter* *aestuarii* sp. nov. is proposed. The type strain is H1-1-2A^T^ (= MCCC 1K04284^T^ =KCTC 82672^T^); ZN11-R3-1 (=MCCC 1A17971=KCTC 82671) is the second strain of the species. Based on the phylogenetic analysis, four novel genera within the family *Zoogloeaceae*, *Parazoarcus* gen. nov., *Pseudothauera* gen. nov., *Pseudazoarcus* gen. nov., and *Cognatazoarcus* gen. nov. were proposed.

**Description of** ***Nitrogeniibacter aestuarii* sp. nov.**

*Nitrogeniibacter aestuarii* (aes.tu.a’ri.i. L. gen. n. *aestuarii*, of a coastal wetland, the source of the type strain isolated from wetland cordgrass and mangrove in estuary).

Colonies on MB agar plates cultured for 3 days at 30 ^o^C are ~1 mm, round, transparent and convex. Cells are Gram-stain-negative and rod-shaped. Growth occurs between 15 and 40 ^o^C with an optimum at 35 ^o^C, at 0-4% NaCl (w/v) with an optimum of 0.5%, and a pH range of 7.0-8.0. Catalase-positive and oxidase-positive. Strains can grow on nitrogen-free medium. Nitrate cannot be reduced to nitrite. Positive for alkaline phosphatase, leucine arylamidase; weakly positive for esterase (C4), valine arylamidase, acid phosphatase, and naphtholAS-BI-phosphohydrolase. Hydrolysis of aesculin is weak positive. Malic acid and trisodium citrate can be used as sole carbon sources.

The quinone system is ubiquinone-8. The major fatty acids are summed feature 3 (C_16:1_*ω*7*c* and C_16:1_*ω*6*c*), C_16:0_, C_12:0_, and C_10:0_ 3-OH. The major polar lipids include phosphatidylethanolamine (PE), diphosphatidylglycerol (DPG), and phosphatidylglycerol (PG). The genome size is 4.7 Mbp with DNA G+C content of 62.7%.

The type strain is H1-1-2A^T^ (= MCCC 1K04284^T^ = KCTC 82672^T^), isolated from *Spartina alterniflora* wetland sediment. Another strain is ZN11-R3-1 (=MCCC 1A17971=KCTC 82671), isolated from the enrichment culture inoculated with plastics collected from a wetland mangrove.

The GenBank/EMBL/DDBJ accession numbers of 16S rRNA gene sequence of strains H1-1-2A^T^ and ZN11-R3-1 are MW644766 and MW644767, respectively. The whole genome sequences of strains H1-1-2A^T^ and ZN11-R3-1 have been deposited at GenBank under the accession numbers CP071321-CP071322 and JAFKAB000000000, respectively.

**Emended Description of the Family *Zoogloeaceae* Boden *et al*. 2017**

In addition to the properties listed in the original description ([Boden et al., 2017](#_ENREF_2)), the family *Zoogloeaceae* includes the genera *Niveibacterium*, *Parazoarcus*, *Pseudothauera*, *Pseudazoarcus*, and *Cognatazoarcus*. The AAI values among the members range from 60.34% to 94.53%. DNA G+C content is 56.6-68.7%.

**Taxonomic Consequences: New Genera**

**Description of *Pseudazoarcus* gen. nov.**

*Pseudazoarcus* (Pseuda.zo.ar’cus. Gr. masc. adj. *pseudes*, false; N.L. masc. n. *Azoarcus*, a bacterial genus name; N.L. masc. n. *Pseudazoarcus*, false *Azoarcus*).

The description is as that for *Pseudazoarcus pumilus* comb. nov., which is the type species. The genus has been separated from *Azoarcus* based on phylogenetic analyses of 16S rRNA gene and genome sequences. The genomic size is 3.2-4.2 Mb. DNA G+C content is 62.8-66.5%.

**Description of *Pseudothauera* gen. nov.**

*Pseudothauera* (Pseu.do.thau’e.ra. Gr. masc. adj. *pseudes*, false; N.L. fem. n. *Thauera*, a bacterial genus name; N.L. fem. n. *Pseudothauera*, false *Thauera*).

The description is as that for *Pseudothauera hydrothermalis* comb. nov., which is the type species. The genus has been separated from *Thauera* based on phylogenetic analysis of 16S rRNA gene sequence and genome sequences. The genomic size is 3.1 Mb-4.7 Mb. DNA G+C content is 63.4-68.3%.

**Description of *Cognatazoarcus* gen. nov.**

*Cognatazoarcus* (Cog.nat.a.zo.ar’cus. L. masc. adj. *cognatus*, relative, related, kindred; N.L. masc. n. *Azoarcus*, a bacterial generic name; N.L. masc. n. *Cognatazoarcus*, related to *Azoarcus*)

The description is as that for *Cognatazoarcus halotolerans* comb. nov., which is the type species. The genus has been separated from *Azoarcus* based on phylogenetic analysis of genome sequences.

**Description of *Parazoarcus* gen. nov.**

*Parazoarcus (*Para.zo.ar'cus. Gr. prep. *para* beside; N.L. masc. n. *Azoarcus*, a bacterial genus name; N.L. masc. n. *Parazoarcus*, beside *Azoarcus*).

The description is as that for *Parazoarcus communis* comb. nov., which is the type species. The genus has been separated from *Azoarcus* based on phylogenetic analysis of genome sequences.

**Taxonomic Consequences: New Combinations for Species**

**Description of *Pseudazoarcus pumilus* comb. nov.**

*Pseudazoarcus pumilus* (pu’mi.lus. L. masc. adj. *pumilus*, small, tiny)

Basonym: *Azoarcus pumilus* Fu et al. 2019

The description is as for *Azoarcus pumilus* ([Fu et al., 2019](#_ENREF_7)). The type strain is SY39^T^ (=KCTC 62157^T^ =MCCC 1K03430^T^).

**Description of *Pseudothauera hydrothermalis* comb. nov.**

*Pseudothauera hydrothermalis* (hy.dro.ther.ma’lis. Gr. neut. n. *hydor*, water; Gr. masc. adj. *thermos*, hot; N.L. fem. adj. *hydrothermalis*, hydrothermal)

Basonym: *Thauera hydrothermalis* Yang *et al.*, 2018

The description is as for *Thauera hydrothermalis* ([Yang et al., 2018](#_ENREF_39)). The type strain is GD-2^T^ (=NBRC 112472^T^ =CGMCC 1.15527^T^).

**Description of *Pseudothauera lacus* comb. nov.**

*Pseudothauera lacus* (la’cus. L. gen. n. *lacus*, of a lake)

Basonym: *Thauera lacus* Zheng et al. 2019.

The description is as for *Thauera lacus* ([Zheng et al., 2019](#_ENREF_42)). The type strain is D20^T^ (= MCCC 1H00305^T^ =KCTC 62586^T^).

**Description of *Pseudothauera* *rhizosphaerae* comb. nov.**

*Pseudothauera* *rhizosphaerae* (rhi.zo.sphae’rae. Gr. fem. n. *rhiza*, root; Gr. fem. n. *sphaira*, ball, sphere; N.L. gen. n. *rhizosphaerae*, from the rhizosphere)

Basonym: *Azoarcus rhizosphaerae* Lin *et al*. 2020.

The description is as for *Azoarcus rhizosphaerae* ([Lin et al., 2020](#_ENREF_22)). The type strain is CC-YHH848^T^ = BCRC 81060^T^ = JCM 32002^T^)

**Description of *Pseudothauera* *nasutitermitis* comb. nov.**

*Pseudothauera* *nasutitermitis* (na.su.ti.ter’mi.tis. N.L. gen. n. *nasutitermitis*, of a termite of the genus *Nasutitermes*)

Basonym: *Azoarcus nasutitermitis* Lin *et al*. 2020.

The description is as for *Azoarcus nasutitermitis* ([Lin et al., 2020](#_ENREF_22)). The type strain is CC-YHH838^T^ (= BCRC 81059^T^ = JCM 32001^T^).

**Description of *Cognatazoarcus halotolerans* comb. nov.**

*Cognatazoarcus halotolerans* (ha.lo.to’le.rans. Gr. masc. n. *hals*, *halos*, salt; L. pres. part. *tolerans*, tolerating; N.L. part. adj. *halotolerans*, salt-tolerating)

Basonym: *Azoarcus halotolerans* Li et al. 2020

The description is as for *Azoarcus halotolerans* ([Li et al., 2020](#_ENREF_20)). The type strain is HKLI-1^T^ (= KCTC 72659^T^ =CCTCC AB 2019312^T^).

**Description of *Parazoarcus communis* comb. nov.**

*Parazoarcus communis* (com.mu’nis. L. masc. adj. *communis*, usual, common, referring to diverse habitats)

Basonym: *Azoarcus communis* Reinhold-Hurek et al. 1993

The description is as for *Azoarcus communis* ([Reinhold-Hurek et al., 1993](#_ENREF_33)). The type strain is SWub3^T^ (= ATCC 51397^T^ = DSM 12120^T^ = LMG 9095^T^).

**Conflicts of interest**

The authors declare no conflicts of interest.

**Author Contributions**

Z. Huang and Z. Shao conceived the study. Z. Huang, R. Liu, F. Chen and Qiliang Lai conducted the experiments. A. Oren proposed names, wrote, and checked etymologies, and edited and corrected the manuscript. Z. Huang and Z. Shao wrote the manuscript.

**Acknowledgements**

This work was supported by marine microbial collection program (2019KJ25) as part of the National Infrastructure of Microbial Resources of China (NIMR 2021-9) and Scientific Research Foundation of Third Institute of Oceanography, Ministry of Natural Resources (2019021).

**References**

Aziz, R.K., Bartels, D., Best, A.A., DeJongh, M., Disz, T., Edwards, R.A., et al. (2008). The RAST Server: rapid annotations using subsystems technology. *BMC Genomics* 9**,** 75. doi: 10.1186/1471-2164-9-75.

Boden, R., Hutt, L.P., and Rae, A.W. (2017). Reclassification of *Thiobacillus aquaesulis* (Wood & Kelly, 1995) as *Annwoodia aquaesulis* gen. nov., comb. nov., transfer of *Thiobacillus* (Beijerinck, 1904) from the *Hydrogenophilales* to the *Nitrosomonadales*, proposal of *Hydrogenophilalia* class. nov. within the '*Proteobacteria*', and four new families within the orders *Nitrosomonadales* and *Rhodocyclales*. *Int J Syst Evol Microbiol* 67(5)**,** 1191-1205. doi: 10.1099/ijsem.0.001927.

Bowers, R.M., Kyrpides, N.C., Stepanauskas, R., Harmon-Smith, M., Doud, D., Reddy, T.B.K., et al. (2017). Minimum information about a single amplified genome (MISAG) and a metagenome-assembled genome (MIMAG) of bacteria and archaea. *Nat Biotechnol* 35(8)**,** 725-731. doi: 10.1038/nbt.3893.

Camacho, C., Coulouris, G., Avagyan, V., Ma, N., Papadopoulos, J., Bealer, K., et al. (2009). BLAST+: architecture and applications. *BMC Bioinformatics* 10(1)**,** 421-429. doi: 10.1186/1471-2105-10-421.

Chaumeil, P.A., Mussig, A.J., Hugenholtz, P., and Parks, D.H. (2019). GTDB-Tk: a toolkit to classify genomes with the Genome Taxonomy Database. *Bioinformatics*. doi: 10.1093/bioinformatics/btz848.

Chun, J., Young Kang, J., Cheol Jung, Y., and Yeop Jahng, K. (2016). *Niveibacterium umoris* gen. nov., sp. nov., isolated from wetland freshwater. *Int J Syst Evol Microbiol* 66(2)**,** 997-1002. doi: 10.1099/ijsem.0.000826.

Fu, G.Y., Yu, X.Y., Yu, X.D., Zhao, Z., Chen, C., Wang, R.J., et al. (2019). *Azoarcus pumilus* sp. nov., isolated from seawater in Sanya, China. *Int J Syst Evol Microbiol* 69(5)**,** 1459-1464. doi: 10.1099/ijsem.0.003341.

Gittel, A., Kofoed, M.V., Sørensen, K.B., Ingvorsen, K., and Schramm, A. (2012). Succession of *Deferribacteres* and *Epsilonproteobacteria* through a nitrate-treated high-temperature oil production facility. *Syst Appl Microbiol* 35(3)**,** 165-174. doi: 10.1016/j.syapm.2012.01.003.

Gurevich, A., Saveliev, V., Vyahhi, N., and Tesler, G. (2013). QUAST: quality assessment tool for genome assemblies. *Bioinformatics* 29(8)**,** 1072-1075. doi: 10.1093/bioinformatics/btt086.

Huang, X.F., Liu, Y.J., Dong, J.D., Qu, L.Y., Zhang, Y.Y., Wang, F.Z., et al. (2014). *Mangrovibacterium diazotrophicum* gen. nov., sp. nov., a nitrogen-fixing bacterium isolated from a mangrove sediment, and proposal of *Prolixibacteraceae* fam. nov. *Int J Syst Evol Microbiol* 64(Pt 3)**,** 875-881. doi: 10.1099/ijs.0.052779-0.

Huang, Z., Dong, L., Lai, Q., and Liu, J. (2020a). *Spartinivicinus ruber* gen. nov., sp. nov., a novel marine gammaproteobacterium producing heptylprodigiosin and cycloheptylprodigiosin as major red pigments. *Front Microbiol* 11**,** 2056. doi: 10.3389/fmicb.2020.02056.

Huang, Z., Du, Y., Lai, Q., and Shao, Z. (2019). *Nonlabens xiamenensis* sp. nov., isolated from coastal seawater. *Antonie van Leeuwenhoek* 112(8)**,** 1263-1271. doi: 10.1007/s10482-019-01258-8.

Huang, Z., Hu, Y., Lai, Q., and Guo, Y. (2020b). Description of *Maribellus sediminis* sp. nov., a marine nitrogen-fixing bacterium isolated from sediment of cordgrass and mangrove. *Syst Appl Microbiol* 43(4)**,** 126099. doi: 10.1016/j.syapm.2020.126099.

Kim, M., Oh, H.S., Park, S.C., and Chun, J. (2014). Towards a taxonomic coherence between average nucleotide identity and 16S rRNA gene sequence similarity for species demarcation of prokaryotes. *Int J Syst Evol Microbiol* 64(Pt 2)**,** 346-351. doi: 10.1099/ijs.0.059774-0.

Konstantinidis, K.T., Rosselló-Móra, R., and Amann, R. (2017). Uncultivated microbes in need of their own taxonomy. *ISME J***,** 2399-2406. doi: 10.1038/ismej.2017.113.

Lagesen, K., Hallin, P., Rodland, E.A., Staerfeldt, H.H., Rognes, T., and Ussery, D.W. (2007). RNAmmer: consistent and rapid annotation of ribosomal RNA genes. *Nucleic Acids Res* 35(9)**,** 3100-3108. doi: 10.1093/nar/gkm160.

Lapidus, A.L., and Korobeynikov, A.I. (2021). Metagenomic data assembly - the way of decoding unknown microorganisms. *Front Microbiol* 12**,** 613791. doi: 10.3389/fmicb.2021.613791.

Lee, D.J., Wong, B.T., and Adav, S.S. (2014). *Azoarcus taiwanensis* sp. nov., a denitrifying species isolated from a hot spring. *Appl Microbiol Biotechnol* 98(3)**,** 1301-1307. doi: 10.1007/s00253-013-4976-9.

Letunic, I., and Bork, P. (2007). Interactive Tree Of Life (iTOL): an online tool for phylogenetic tree display and annotation. *Bioinformatics* 23(1)**,** 127-128. doi: 10.1093/bioinformatics/btl529.

Li, S., Zhao, L., Han, J., Liu, S., Dai, J., Fu, G., et al. (2020). *Azoarcus halotolerans* sp. nov., a novel member of *Rhodocyclaceae* isolated from activated sludge collected in Hong Kong. *Int J Syst Evol Microbiol* 70(11)**,** 5799-5805. doi: 10.1099/ijsem.0.004476.

Liao, H., Qu, M., Hou, X., Lin, X., Li, H., Duan, C.S., et al. (2021). *Nitrogeniibacter mangrovi* gen. nov., sp. nov., a novel anaerobic and aerobic denitrifying betaproteobacterium and reclassification of *Azoarcus pumilus* as *Aromatoleum pumilum* comb. nov. *Int J Syst Evol Microbiol* 71(8). doi: 10.1099/ijsem.0.004946.

Lin, S.Y., Hameed, A., Tsai, C.F., Huang, G.H., Hsu, Y.H., and Young, C.C. (2020). Description of *Azoarcus nasutitermitis* sp. nov. and *Azoarcus rhizosphaerae* sp. nov., two nitrogen-fixing species isolated from termite nest and rhizosphere of *Ficus religiosa*. *Antonie van Leeuwenhoek* 113(7)**,** 933-946. doi: 10.1007/s10482-020-01401-w.

Liu, B., Mao, Y., Bergaust, L., Bakken, L.R., and Frostegard, A. (2013). Strains in the genus *Thauera* exhibit remarkably different denitrification regulatory phenotypes. *Environ Microbiol* 15(10)**,** 2816-2828. doi: 10.1111/1462-2920.12142.

Luo, C., Rodriguez, R.L., and Konstantinidis, K.T. (2014). MyTaxa: an advanced taxonomic classifier for genomic and metagenomic sequences. *Nucleic Acids Res* 42(8)**,** e73. doi: 10.1093/nar/gku169.

Macy, J.M., Rech, S., Auling, G., Dorsch, M., Stackebrandt, E., and Sly, L.I. (1993). *Thauera selenatis* gen. nov., sp. nov., a member of the beta subclass of *Proteobacteria* with a novel type of anaerobic respiration. *Int J Syst Bacteriol* 43(1)**,** 135-142. doi: 10.1099/00207713-43-1-135.

Mechichi, T., Stackebrandt, E., Gad'on, N., and Fuchs, G. (2002). Phylogenetic and metabolic diversity of bacteria degrading aromatic compounds under denitrifying conditions, and description of *Thauera phenylacetica* sp. nov., *Thauera aminoaromatica* sp. nov., and *Azoarcus buckelii* sp. nov. *Arch Microbiol* 178(1)**,** 26-35. doi: 10.1007/s00203-002-0422-6.

Orata, F.D., Meier-Kolthoff, J.P., Sauvageau, D., and Stein, L.Y. (2018). Phylogenomic Analysis of the Gammaproteobacterial Methanotrophs (Order Methylococcales) Calls for the Reclassification of Members at the Genus and Species Levels. *Front Microbiol* 9**,** 3162. doi: 10.3389/fmicb.2018.03162.

Parks, D.H., Chuvochina, M., Waite, D.W., Rinke, C., Skarshewski, A., Chaumeil, P.A., et al. (2018). A standardized bacterial taxonomy based on genome phylogeny substantially revises the tree of life. *Nat Biotechnol* 36(10)**,** 996-1004. doi: 10.1038/nbt.4229.

Parks, D.H., Imelfort, M., Skennerton, C.T., Hugenholtz, P., and Tyson, G.W. (2015). CheckM: assessing the quality of microbial genomes recovered from isolates, single cells, and metagenomes. *Genome Res* 25(7)**,** 1043-1055. doi: 10.1101/gr.186072.114.

Qin, Q.L., Xie, B.B., Zhang, X.Y., Chen, X.L., Zhou, B.C., Zhou, J., et al. (2014). A proposed genus boundary for the prokaryotes based on genomic insights. *J Bacteriol* 196(12)**,** 2210-2215. doi: 10.1128/JB.01688-14.

Rabus, R., Wohlbrand, L., Thies, D., Meyer, M., Reinhold-Hurek, B., and Kämpfer, P. (2019). *Aromatoleum* gen. nov., a novel genus accommodating the phylogenetic lineage including *Azoarcus evansii* and related species, and proposal of *Aromatoleum aromaticum* sp. nov., *Aromatoleum petrolei* sp. nov., *Aromatoleum bremense* sp. nov., *Aromatoleum toluolicum* sp. nov. and *Aromatoleum diolicum* sp. nov. *Int J Syst Evol Microbiol* 69(4)**,** 982-997. doi: 10.1099/ijsem.0.003244.

Rai, A., Jagadeeshwari, U., Deepshikha, G., Smita, N., Sasikala, C., and Ramana, C.V. (2021). Phylotaxogenomics for the Reappraisal of the Genus Roseomonas With the Creation of Six New Genera. *Front Microbiol* 12**,** 677842. doi: 10.3389/fmicb.2021.677842.

Reinhold-Hurek, B., Hurek, T., Gillis, M., Hoste, B., Vancanneyt, M., Kersters, K., et al. (1993). *Azoarcus* gen. nov., nitrogen-fixing *Proteobacteria* associated with roots of Kallar grass (*Leptochloa fusca* (L.) Kunth), and description of two species, *Azoarcus indigens* sp. nov. and *Azoarcus communis* sp. nov. *Int J Syst Bacteriol* 43(3)**,** 574-584. doi: doi.org/10.1099/00207713-43-3-574.

Rinke, C., Schwientek, P., Sczyrba, A., Ivanova, N.N., Anderson, I.J., Cheng, J.F., et al. (2013). Insights into the phylogeny and coding potential of microbial dark matter. *Nature* 499(7459)**,** 431-437. doi: 10.1038/nature12352.

Shin, Y.K., Hiraishi, A., and Sugiyama, J. (1993). Molecular systematics of the genus *Zoogloea* and emendation of the genus. *Int J Syst Bacteriol* 43(4)**,** 826-831. doi: 10.1099/00207713-43-4-826.

Suresh, G., Lodha, T.D., Indu, B., Sasikala, C., and Ramana, C.V. (2019). Taxogenomics Resolves Conflict in the Genus *Rhodobacter*: A Two and Half Decades Pending Thought to Reclassify the Genus *Rhodobacter*. *Front Microbiol* 10**,** 2480. doi: 10.3389/fmicb.2019.02480.

Wang, Z., Li, W., Li, H., Zheng, W., and Guo, F. (2020). Phylogenomics of *Rhodocyclales* and its distribution in wastewater treatment systems. *Sci Rep* 10(1)**,** 3883. doi: 10.1038/s41598-020-60723-x.

Weon, H.Y., Kim, B.Y., Yoo, S.H., Kwon, S.W., Go, S.J., and Stackebrandt, E. (2008). *Uliginosibacterium gangwonense* gen. nov., sp. nov., isolated from a wetland, Yongneup, in Korea. *Int J Syst Evol Microbiol* 58(1)**,** 131-135. doi: 10.1099/ijs.0.64567-0.

Yang, L., Muhadesi, J.B., Wang, M.M., Wang, B.J., Liu, S.J., and Jiang, C.Y. (2018). *Thauera hydrothermalis* sp. nov., a thermophilic bacterium isolated from hot spring. *Int J Syst Evol Microbiol* 68(10)**,** 3163-3168. doi: 10.1099/ijsem.0.002960.

Yoon, S.H., Ha, S.M., Kwon, S., Lim, J., Kim, Y., Seo, H., et al. (2017a). Introducing EzBioCloud: a taxonomically united database of 16S rRNA gene sequences and whole-genome assemblies. *Int J Syst Evol Microbiol* 67(5)**,** 1613-1617. doi: 10.1099/ijsem.0.001755.

Yoon, S.H., Ha, S.M., Lim, J., Kwon, S., and Chun, J. (2017b). A large-scale evaluation of algorithms to calculate average nucleotide identity. *Antonie van Leeuwenhoek* 110(10)**,** 1281-1286. doi: 10.1007/s10482-017-0844-4.

Zheng, W.S., Liang, Q.Y., and Du, Z.J. (2019). *Thauera lacus* sp. nov., isolated from a saline lake in Inner Mongolia. *Int J Syst Evol Microbiol* 69(12)**,** 3786-3791. doi: 10.1099/ijsem.0.003679.

**Table 1**. Differential characteristics of strain H1-1-2A^T^ and strain ZN11-R3-1 compared to the close relative *N. mangrovi* M9-3-2^T^.

All strains were positive for leucine arylamidase, weak positive for esterase (C4), valine arylamidase, acid phosphatase, naphthol-AS-BI-phosphohydrolase. Hydrolysis of aesculin were weak positive. +, positive; w, weak positive; -, negative.

| Characteristics | H1-1-2A^T^ | ZN11-R3-1 | M9-3-2^T^ |
| --- | --- | --- | --- |
| Temperature (optimum, ^o^C) | 15-40 (35) | 15-40 (35) | 25-40 (35) |
| pH | 7-8 | 7-8 | 7 |
| Alkaline phosphatase | + | + | w |
| Lipase(C14) | - | - | w |
| Reduction of nitrate to nitrite | - | - | + |
| Trisodium citrate as sole carbon source for growth | + | + | - |
| Voges–Proskauer reaction | - | - | w |
| Genome size (bp, >1 kb) | 4,745,026 | 4,656,485 | 4,236,644 |
| Functional genes | 4,364 | 4,317 | 3,884 |
| DNA G+C content (%) | 62.67 | 62.71 | 67.13 |
| Isolation source | *Spartina alterniflora* sediment | Plastics in mangrove sediment | Mangrove sediment |

**Table 2.** Fatty acid profile of strain H1-1-2A^T^ and strain ZN11-R3-1 compared to close relative *N. mangrovi* M9-3-2^T^.

-, not detected; tr, trace (<1%).

| Fatty acids | H1-1-2A | ZN11-R3-1 | M9-3-2 |
| --- | --- | --- | --- |
| Saturated |  |  |  |
| C_9:0_ | 2.0 | tr | 1.1 |
| C_10:0_ | 2.7 | 1.0 | 2.3 |
| C_12:0_ | 9.5 | 6.3 | 2.6 |
| C_14:0_ | - | tr | 5.1 |
| C_16:0_ | 23.0 | 26.1 | 34.0 |
| C_17:0_ | 1.5 | 1.2 | 1.8 |
| C_18:0_ | tr | 2.0 | 6.4 |
| Unsaturated |  |  |  |
| iso-C_17:1_ *ω*5*c* | 1.0 | tr | 1.1 |
| C_17:1_ *ω*6*c* | 1.1 | tr | - |
| anteiso-C_17:1_ A | - | 1.0 | tr |
| iso-C_18:1_ H | 1.2 | tr | 1.1 |
| C_18:1_ *ω*9*c* | - | tr | 1.6 |
| Branched |  |  |  |
| iso-C_12:0_ | - | 2.1 | tr |
| Hydroxyl |  |  |  |
| C_10:0_ 3-OH | 7.6 | 5.1 | 5.0 |
| Summed feature 2† | - | tr | 1.7 |
| Summed feature 3† | 43.2 | 37.9 | 28.7 |
| Summed feature 8† | 4.7 | 8.7 | 4.7 |

†Summed features are groups of two or three fatty acids that cannot be separated by GLC using the MIDI system. summed feature 2 comprised C_12:0_ aldehyde and unknown 10.9283, summed feature 3 comprised C_16:1_*ω*7*c* and C_16:1_*ω*6*c*, and summed feature 8 comprised C_18:1_ *ω*7*c* and/or C_18:1_ *ω*6*c*.

**Figure legend**

**Figure 1**. Phylogeny of 16S rRNA gene sequences. The tree was constructed using the neighbor joining method. Bootstrapping was carried out with 1,000 replicates. Branch node values below 70% are not shown. *Burkholderia cepacia* ATCC 25416^T^ (AXBO01000009) was selected as the outgroup. Bar, 0.05 represented the nucleotide substitution per position. Members of *Nitrogeniibacter*, *Pseudazoarcus* and *Pseudothauera* are marked bold.

**Figure 2**. Phylogenomic analysis based on 120 bacterial covered single-copied gene sets of the members affiliated to the order *Rhodocyclales* using FastTree. The bootstrap values on the node are displayed by >70. Bar, 0.1 represents the nucleotide substitutions per position. The blue name represents the validly published species. Red names showed *Nitrogeniibacter* members. The genus names were shown around the color circle. Four genera proposed in this study are marked bold.

**Figure 3**. Phylogenomic analysis of the *Nitrogeniibacter* members and type strains affiliated to the order *Rhodocyclales* based on 120 bacterial conserved single-copied gene sets of the members. The bootstrap values on the nodes are displayed by >70. Bar, 0.1 represents the nucleotide substitution per position. Blue circles represent type species. Red names show *Nitrogeniibacter* members. The branch color represents the families, *Zoogloeaceae*, *Azonexaceae* and *Rhodocyclaceae* of the order *Rhodocyclales*. Four genera proposed in this study are marked bold.

**Figure 4**. Heatmap showing the AAI values (upper panel) and POCP values (lower panel) among the members of the family *Zoogloeaceae*.

**Supplementary Materials**

**Supplementary Figure 1**. Electrophoresis pattern of BOX-PCR fingerprinting of the strains.

M. DL 2000 DNA marker; 1, strain M9-3-2^T^; 2, strain H1-1-2A^T^; 3. strain ZN11-R3-1.

**Supplementary Figure 2.** Phylogeny of 16S rRNA gene sequences. The tree was constructed using the maximum likelihood method. Bootstrapping was carried out with 1000 replicates. Branch node values below 70% are not shown. *Burkholderia cepacia* ATCC 25416^T^ (AXBO01000009) was selected as the outgroup. Bar, 0.05 represent the nucleotide substitutions per position.

**Supplementary Figure 3**. Polar lipid profile of strain H1-1-2A^T^.

Abbreviations: PE, phosphatidylethanolamine; DPG, diphosphatidylglycerol, and PG, phosphatidylglycerol, APL, aminophospholipid, PL, unidentified phospholipid; L, unidentified lipid.

**Supplementary Table 1.** Nitrogen-fixation genes predicted from the genome of strain H1-1-2A^T^.

**Supplementary Table 2.** Denitrification genes predicted from the genome of strain M9-3-2^T^.

**Supplementary Table 3.** Sulfur oxidation genes found in the genome of strain H1-1-2A^T^.

**Supplementary Table 4.** Genomic features of the members affiliated to the family *Zoogloeaceae*.

**Supplementary Table 5**. Comparison of chemotaxonomic characteristics among the closely related genera.
